# *Brucella ligniniphila* sp. nov., a Novel Lignin-Degrading Bacterium from Decaying Bamboo, and Synergistic Lignin Biodegradation by Consortium with White-Rot Fungi

**DOI:** 10.3390/microorganisms14091923

**Published:** 2026-08-31

**Authors:** Shuaibo Han, Xiaolong He, Jianglong Guo, Jinping Deng, Xueye Li, Jiayin Wang, Xinxing Wu, Hui Wang, Yan Zhang, Fangli Sun

**Affiliations:** 1 National Engineering & Technology Research Center of Wood-Based Resources Comprehensive Utilization, School of Chemical and Materials Engineering, Zhejiang A&F University, Hangzhou 311300, China; 2 Microbes and Insects Control Institute of Bio-Based Materials, Zhejiang A&F University, Hangzhou 311300, China; 3 Zhejiang Provincial Key Laboratory of Germplasm Innovation and Utilization for Garden Plants, School of Landscape and Architecture, Zhejiang A&F University, Hangzhou 311300, China

**Keywords:** lignin degradation, *Brucella*, GC-MS analysis, genomic analysis, bacterial–fungal co-culture

## Abstract

A lignin-degrading bacterium, strain BE17^T^, was isolated from decaying bamboo and identified as a novel species within the genus *Brucella*, proposed as *Brucella ligniniphila*, sp. nov. (type strain = MCCC 1K08845^T^ = JCM 36600^T^). BE17^T^ exhibited a lignin degradation rate of 7.9% with lignin as the sole carbon source, which increased to 17.2% upon glucose supplementation. Structural analysis indicated that BE17^T^ degraded lignin by cleaving β-O-4 bonds, oxidizing aliphatic side chains and disrupting aromatic rings. Low-molecular-weight lignin fractions were more readily degraded. Genome analysis revealed genes encoding DyPs, peroxidases, monooxygenases, and dioxygenases. GC-MS analysis identified aromatic monomers and organic acids among the degradation products, and together with genome annotation, these data suggested the involvement of CoA-dependent non-β-oxidative, β-ketoadipate, and phenylacetic acid pathways. To enhance lignin degradation efficiency, a co-culture system (BT) of BE17^T^ and *Trametes versicolor* was established. This system achieved a degradation rate of 36.7% under optimized conditions (25 °C, pH 6.0, BE17^T^ inoculated on day 9). Microstructural and chemical analyses confirmed that fungal pretreatment facilitated lignin degradation. This study reveals the lignin-degrading potential of a novel *Brucella* species and demonstrates the synergistic effect of a bacterial–fungal system, offering insights into lignin biodegradation and bioconversion strategies.

## 1. Introduction

Fast-growing greenhouse gas emissions and limited fossil fuel reserves have spurred the development of sustainable biorefinery technologies based on renewable feedstocks [1]. Lignocellulosic biomass, which comprises cellulose, hemicellulose and lignin, is the most adequate plant biomass and offers a low-cost source of fermentable sugars and building blocks for biofuels and bioproducts [2]. It is considered a carbon-neutral alternative to petroleum-derived chemicals, capable of being converted into bioethanol, bioenergy and a variety of green chemicals.

As the second most prevalent biopolymer on the planet following cellulose, lignin constitutes 10–35% of lignocellulosic biomass [3,4]. It is synthesized by oxidative coupling of three monolignols (sinapyl, coniferyl and p-coumaryl alcohols), yielding aromatic units known as syringyl (S), guaiacyl (G), and p-hydroxyphenyl (H) substructures [5,6]. It is a complex aromatic polymer that provides structural stiffness to plant cell walls and confers resistance against pathogens [7]. However, its recalcitrant three-dimensional matrix hinders enzymatic access to polysaccharides, thereby limiting the efficient conversion of lignocellulose [8]. Overcoming lignin’s resistance to breakdown is, therefore, a key challenge for biomass conversion. Efficient lignin deconstruction is critically important not only for releasing cellulosic sugars but also for producing valuable aromatic chemicals and for carbon cycling in the environment [9].

Conventional lignin-removal methods typically rely on harsh chemical or thermochemical pretreatments. Strong acids or bases, ionic liquids, and metal-catalyzed oxidations at high temperature and pressure can break down lignin polymers [10]. These processes are energy-intensive and may lead to serious environmental problems. In contrast, microbial degradation of lignin under mild conditions has attracted increasing interest and been viewed as an attractive route for lignin valorization [11]. In nature, a variety of microorganisms participate in lignin degradation. White-rot fungi have been extensively studied for their potent ligninolytic systems, which employ extracellular enzymes such as lignin peroxidases (LiPs), manganese peroxidases (MnPs), and laccases (Lacs) [12]. These fungal systems are capable of degrading lignin to carbon dioxide and small organics [13].

Compared with fungi, bacteria offer several advantages, including smaller genome size, greater ease of genetic manipulation, and more straightforward large-scale recombinant expression of important enzymes [14]. These characteristics have led to a renewed focus on bacteria for the discovery of novel strains and enzymes involved in lignin degradation [15]. While lignin degradation by fungus has received a lot of attention, reports of lignin-degrading capabilities in bacteria are few. Molecular techniques have been used to study catabolic processes in some bacteria that include genes, enzymes, and lignin derivatives. However, it is important to note that lignin degradation mechanisms vary significantly across bacterial species. In *Sphingobacterium* sp. HY-H, lignin is first broken down into molecules with low molecular weight [16]. *Sphingomonas paucimobilis* SYK-6 produces the corresponding ketone by oxidizing the hydroxyl group of β-aryl ether lignin compounds [17]. In the absence of hydrogen peroxide, *Rhodococcus jostii* RHA1 degrades lignin via the β-ketoadipate pathway (β-KAP), an enzyme-mediated route for cleaving aromatic rings [18]. Despite these findings, many metabolic pathways of lignin and its derivatives remain unclear across diverse bacterial species. Finding new ligninolytic microorganisms and enzymes and characterizing them biochemically will facilitate the breakdown of biomass for use in the biofuel and bioproduct industries [14].

The genus *Brucella*, within the family *Brucellaceae*, includes *Brucella melitensis* as its type species and was first described by Meyer and Shaw in 1920 [19]. Currently, there are over 30 recognized species in the genus *Brucella* (https://lpsn.dsmz.de/genus/brucella, accessed on 30 June 2026), which are isolated mainly from humans and animals [19,20,21,22,23]. Generally, *Brucella* is regarded as an intracellular, facultative extracellular pathogen able to colonize phagocytic cells to evade the host’s adaptive immune system, multiply, and spread throughout the organism [24]. However, recent study has found that some members of *Brucella* show a high capacity for degrading refractory organics, such as polyethylene terephthalate [25], phthalate esters [26], organophosphorus pesticides [27], and natural rubber [28]. It has been reported that the *Brucella* genus contains phenol-degrading species that can effectively decompose phenol via catechol 1,2-dioxygenase [29]. *Brucella suis* exhibits the genetic and proteomic characteristics of the β-ketoadipate route and the homoprotocatechuate pathway, which are essential metabolic pathways for the degradation of aromatic chemicals in prokaryotes [30]. Nevertheless, the contribution of *Brucella* to lignin degradation remains relatively underexplored.

Given the fact that bacterial lignin degradation is generally less efficient than that of write rot fungi due to weaker extracellular enzyme secretion ability [31,32], co-culture systems were created, which are derived from natural microbial consortia under regulated and simplified conditions, incorporate a minimum of two constituents, and exploit biological interactions such as competition, cooperation, and elicitation among members [33]. Bacteria–fungi co-cultures have been extensively documented to improve bioremediation efficacy compared with monoculture. According to Alisi et al. [34], microbial consortia’s multi-component systems offer a broad metabolic network that may get beyond the drawbacks of using only one microbial species in bioremediation procedures and guarantee more thorough biodegradation. Li and Li [35] discovered that the use of fungus–bacteria consortia for the biodegradation of petroleum hydrocarbons was more effective than the total of the removals from pure cultures of bacteria and fungi. Zhao et al. [36] demonstrated that bacterial and fungal co-culture consortia significantly increase the effectiveness of alkaline lignin degradation.

Bamboo, which possesses prominent physical and environmental features, is extensively employed in the building and furniture industries [37]. In recent years, bamboo has acquired increased significance as an alternative material to wood owing to the global scarceness of forest resources. However, bamboo is particularly susceptible to mold and decay caused by microbial activity, which impacts its aesthetics and durability [38,39,40]. Bamboo lignocellulose is characterized by its high lignin content and specific structural features, creating a selective environment for microorganisms with specialized ligninolytic capabilities [41]. Decaying bamboo culms represent a specialized ecological niche where lignin degradation processes are particularly active. The isolation and characterization of novel lignin-degrading bacteria from this habitat could yield valuable insights into lignin biodegradation mechanisms and potentially uncover new enzymes and pathways with biotechnological applications.

**Figure 1:**
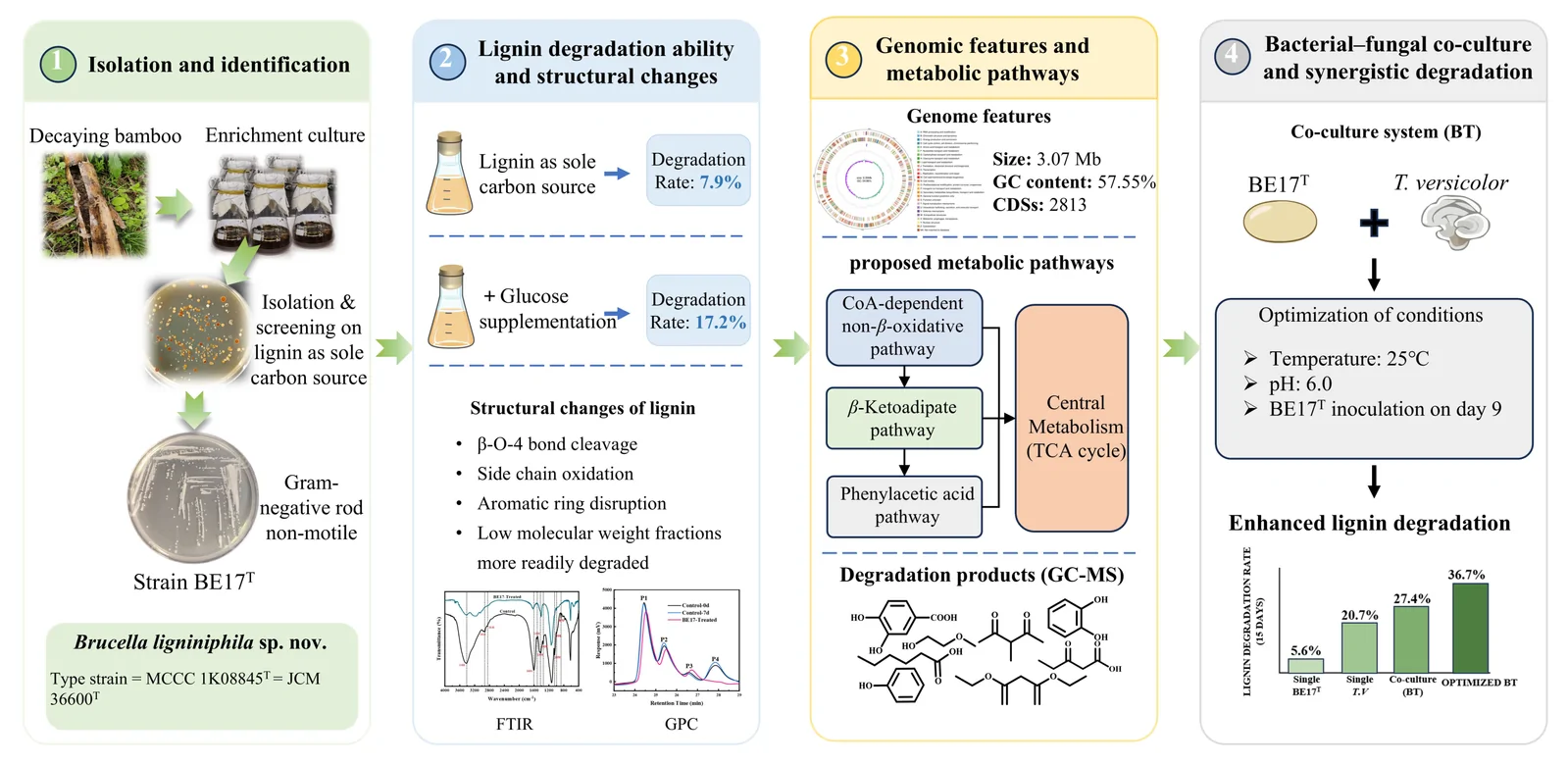
Graphical summary of the experimental workflow. The overall procedure consisted of four main stages: (1) isolation and identification; (2) lignin degradation ability and structural changes; (3) genomic features and metabolic pathways and (4) bacterial–fungal co-culture and synergistic degradation.

In this study, a novel lignin-degrading bacterium belonging to the genus *Brucella* was isolated from decaying bamboo culms. Polyphasic taxonomic analysis revealed that strain BE17^T^ represents a novel species of the genus *Brucella*, for which the name *Brucella ligniniphila* sp. nov. is proposed. Alkaline lignin (AL), which serves as a model compound with a structure analogous to lignocellulose and is frequently utilized as a raw material for lignin degradation research [42], was employed in this work to examine the properties of lignin degradation by strain BE17^T^. Scanning electron microscopy (SEM), Fourier-transform infrared spectroscopy (FTIR), and gel-permeation chromatography (GPC) were employed to investigate the lignin structural changes. Genes involved in lignin degradation were explored via genomic analysis, its breakdown products were determined by GC-MS, and the metabolic pathway of lignin degradation by strain BE17^T^ was further explored through the combination of these two methods. Furthermore, we constructed a co-culture consortium of strain BE17^T^ with the white-rot fungus *Trametes versicolor* and optimized culture conditions to maximize lignin degradation (Figure 1). These results advance our understanding of bacterial lignin biodegradation and highlight the synergistic potential of bacterial–fungal consortia for converting lignocellulosic biomass into valuable products.

## 2. Materials and Methods

### 2.1. Isolation and Cultivation of Strain BE17^T^

Decayed bamboo culms were collected from the campus of Zhejiang A&F University (119°43′37″ E, 30°15′41″ N, 49 m). One gram of decayed bamboo culms was placed in 25 mL of enrichment medium and incubated at 30 °C for 2 days on a shaker set at 150 rpm. The enrichment medium consisted of 5 g of alkaline lignin, 1 g of KH_2_PO_4_, 2 g of (NH_4_)_2_SO_4_, 0.5 g of MgSO_4_, 0.01 g of FeCl_3_, 0.1 g of CaCl_2_, and 1000 mL of distilled water. To inhibit fungal growth, 50 mg/L of nystatin was added to the medium. After enrichment, a 10-fold serial dilution series (10^−1^ to 10^−7^) was prepared using sterile saline. Subsequently, 0.1 mL of each dilution was spread onto primary screening medium plates and incubated at 30 °C for 2–3 days. Colonies exhibiting visible growth on plates with alkaline lignin as the sole carbon source were selected and repeatedly streaked onto fresh screening plates to obtain pure cultures. The purified isolate was subsequently cultivated routinely in Luria–Bertani (LB) broth medium [43] and kept at −80 °C in 25% (*v*/*v*) glycerol for long-term storage and subsequent analyses. For aerobic liquid cultures, tubes and Erlenmeyer flasks were sealed with rubber and cotton plugs, respectively, to allow gas exchange and prevent contamination.

### 2.2. Physiological and Biochemical Characterization

The morphology of the cells was assessed with the use of transmission electron microscopy (JEM-1230, JEOL Ltd., Tokyo, Japan) and an optical microscope (BX40, Olympus, Tokyo, Japan) [44]. Cell motility was assessed using a semisolid LB medium with incubation at 28 °C for 2 weeks [45]. The optimal conditions and acceptable range of salinity, temperature and pH for the growth of strain BE17 was carried out according to the previous study [38]. To assess anaerobic growth, the strain was cultured in modified LB medium placed in an anaerobic jar (Mitsubishi Gas Chemical Company, Inc., Tokyo, Japan) containing an Anaero-Pack based on the method described by Han et al. [46]. The hydrolysis of starch, Tween 20, Tween 40, Tween 60, Tween 80, casein, L-tyrosine, cellulose and tyrosine as well as catalase and oxidase activities were determined according to the method of Sun et al. [47]. Nitrate and nitrite reduction and hydrolysis of CM-cellulose, filter paper, gelatin, xanthine, and hypoxanthine were determined according to our previous study [48]. Additional biochemical assays were conducted following the protocols described by Mata et al. [49]. The Biolog GEN III identification system (Biolog, Hayward, CA, USA) was employed to assess the utilization of individual carbon sources according to the manufacturer’s instructions. Acid production was assessed utilizing API 50CH (bioMérieux, Marcy-l’Étoile, France) strips. Leifson modified O/F medium (MOF) [50] was employed to suspend the cells for the inoculation of API 50CH experiments. Additional enzymatic activities and biochemical properties were evaluated using API 20NE and API ZYM kits (bioMérieux, Marcy-l’Étoile, France), with readings taken after 4 h and 24 h, respectively. Susceptibility to antibiotics was assessed on LB plates for 7 days at 30 °C according to Han et al. [46]. *Brucella gallinifaecis* DSM 15295^T^ was used as a reference strain for comparison under the same condition.

### 2.3. Chemotaxonomic Characterization

The isoprenoid quinone, polar lipid, and fatty acid analyses were performed on BE17^T^ and *B. gallinifaecis* DSM 15295^T^ cells that were cultured on LB medium for three days at 28 °C. Isoprenoid quinones were extracted and purified by thin-layer chromatography (TLC) and were subsequently identified using an Agilent high-performance liquid chromatography–mass spectrometry system (Agilent 1200, Agilent Technologies, Inc., Santa Clara, CA, USA; Thermo Finnigan LCQ DECA XP MAX mass spectrometer, Thermo Fisher Scientific Inc., Waltham, MA, USA) [51]. Two-dimensional TLC was used to examine polar lipids using silica gel 60F254 plates (Merck Millipore, Darmstadt, Germany), as previously mentioned [48]. The two strains were collected and freeze-dried during the exponential stage of development to prepare cellular fatty acid methyl esters (FAMEs), as described by Kuykendall et al. [52]. Cellular fatty acid methyl esters were analyzed using the RTSBA6 database following the protocol of the Microbial Identification System (MIDI; Microbial ID).

### 2.4. Phylogenetic Analysis

Genomic DNA was extracted from strain BE17^T^ using the Bacterial Genome DNA Rapid Extraction Kit (Dongsheng Biotech, Guangzhou, China) based on the manufacturer’s instructions. Primers 27F and 1492R, which are universal bacterial primers, were used to amplify the 16S rRNA gene from genomic DNA [53]. Tsingke Biotechnology Co., Ltd. of Hangzhou, China was entrusted with sequencing the PCR products. The nearly complete 16S rRNA gene sequences obtained from the assembly process were uploaded to EzBioCloud (https://www.ezbiocloud.net) for preliminary taxonomic analysis to determine the evolutionary relationships of the strains [54]. The phylogenetic tree of 16S rRNA gene sequences was constructed using MEGA 11 [55]. Phylogenetic analysis was performed using the Neighbor-Joining (NJ) method [56] with the Kimura two-parameter model [57]. Bootstrap analysis was conducted with 1000 replicates.

### 2.5. Genome Sequencing, Assembly, and Annotation

Whole-genome sequencing was commissioned to Wuhan Benagen Technology Co., Ltd. (Wuhan, China). Bacterial cells in the log phase were harvested using centrifugation, and genomic DNA was isolated with an improved SDS extraction technique. After quality assessment and purification, a one-dimensional library was constructed using the SQK-LSK110 kit (Oxford Nanopore Technologies, Oxford, UK) according to the manufacturer’s instructions. In parallel, a short-fragment library was constructed using the VAHTS^®^ Universal Plus DNA Library Prep Kit for MGI V2/for Illumina V2 (Vazyme, Nanjing, China). Subsequent to library quality control, whole-genome sequencing was conducted using the Nanopore PromethION (Oxford Nanopore Technologies (ONT), Oxford, UK) and Illumina NovaSeq 6000 (Illumina Inc., San Diego, CA, USA) platforms. Sequencing data from those two platforms were assembled using Unicycler v0.5.1 [58]. Plasmid identification was performed using PlasFlow software (Version: 1.1.0–c85355d, https://hub.docker.com/r/nanozoo/plasflow, accessed on 30 June 2026). Gene prediction of the assembled genome was carried out using Prokka (Version: 1.14.6) [59]. The predicted gene sequences were annotated by BLAST+ (Version: 2.11.0+) [60] against several functional databases, including COG [61], GO [62], KEGG [63], and RefSeq. Additional functional annotation was performed using HMMER (Version: 3.3.2) [64] based on the Pfam [65] and TIGERFAM [66] databases. SignalP 6.0 was used to predict the signal peptide of putative ligninolytic enzymes [67]. The average nucleotide identity (ANI) and digital DNA–DNA hybridization (dDDH) were calculated, and corresponding heatmaps were illustrated according to our previous research [68]. A phylogenomic tree was constructed using Type (Strain) Genome Server (https://tygs.dsmz.de) [69].

### 2.6. Biodegradation of Alkaline Lignin by BE17

Strain BE17^T^ was first activated in 3 mL of LB liquid medium in test tubes, then scaled up in 50 mL Erlenmeyer flasks and cultured to the logarithmic growth phase to prepare the working seed culture. The seed culture was centrifuged at 6000 rpm for 5 min, and the cell pellet was resuspended in sterile physiological saline. The cell suspension’s optical density at 600 nm (OD_600_) was set to around 0.8~1.0. Subsequently, 2% (*v*/*v*) of the inoculum was transferred into lignin degradation medium (3 g AL, 2 g (NH_4_)_2_SO_4_, 1 g KH_2_PO_4_, 1 g K_2_HPO_4_, 0.2 g MgSO_4_, 0.05 g FeSO_4_, 0.002 g MnSO_4_, 0.002 g CuSO_4_, 0.1 g NaCl, 0.1 g CaCl_2_, 0.02 g VB_1_ and 0.5 g Tween 80 in 1 L distilled water, pH 7.0) with and without the addition of 1 g/L glucose. Cultures were incubated at 30 °C and 150 rpm for 7 days under shaking conditions. Samples were collected daily to measure biomass and lignin degradation rate.

The growth curve of the strain BE17^T^ was plotted by measuring OD_600_ using the lignin degradation medium without inoculation as the blank control. The supernatant was obtained by centrifuging at 10,000 rpm for 10 min, followed by appropriate dilution and measurement of alkaline lignin concentration using UV spectrophotometry. The alkaline lignin standard curve was established as *y* = 0.005*x* + 0.004, with an *R*^2^ value of 0.999, where *x* represents the alkaline lignin concentration (μg/mL) and *y* represents OD_280_. The lignin degradation rate was calculated using the formula (A_2_ − A_1_)/A_0_, where A_0_ is the initial alkaline lignin concentration after sterilization, A_1_ is the alkaline lignin concentration after a specific period of cultivation with inoculation, and A_2_ is the alkaline lignin concentration after a specific period of cultivation with sterile water as a blank control.

The fermentation broth from lignin degradation was centrifuged at 10,000 rpm for 10 min, and the resulting supernatant was vacuum freeze-dried to obtain a lyophilized powder. After being coated with gold using a sputter coater, the powder was observed under a scanning electron microscope (TM3030, HITACHI, Tokyo, Japan) to detect morphological changes in lignin. Meanwhile, a total of 4 mg of freeze-dried alkaline lignin sample was mixed with 400 mg of dried KBr, and the mixture was ground into a fine powder using an agate mortar. The mixture was then pressed into a uniform thin pellet under a constant pressure of 30 MPa (or 200 kg·cm^−2^) for 2 min using a pellet press. The prepared pellet was placed into a Shimadzu IR spectrometer (IRPrestige-21, HITACHI, Tokyo, Japan) for spectral scanning. A pellet made from pure KBr powder was used as the background reference. The scanning range was set from 4000 cm^−1^ to 400 cm^−1^. The molecular weight of alkaline lignin was determined using GPC as described by Yang et al. [70]. A 5 mL solution of AL in water (20 mg/mL) was introduced onto an Ultrahydrogel™ Linear column of 300 mm × 7.8 mm. The mobile phase consisted of a 0.1 mol/L NaNO_3_ solution with pH of 10.7 at 40 °C, and the flow rate was 1 mL/min. The sample was analyzed using UV-vis spectroscopy (Waters GPC 1515, Leiden ScientificInstruments, Milford, MA, USA) at wavelengths of 254 and 280 nm. Polyethylene (820; 2330; 3680; 13,200; 30,000; 44,000; 570,000; 5,185,000 Da) was used for calibration [71].

### 2.7. Gas Chromatography–Mass Spectrometry (GC-MS) Analysis

Strain BE17^T^ was grown in 50 mL of medium containing alkaline lignin (AL) as the sole carbon source and incubated on a rotary shaker for seven days. The sample was centrifuged at 10,000 rpm for 10 min to remove cells. The following procedure was then used: Adjust the pH of the supernatant to approximately 2.5 using HCl. Extract the supernatant three times with ethyl acetate, dry the extract with anhydrous sodium sulfate, and filter. Concentrate the filtered extract to approximately 10 mL using a rotary evaporator at 37 °C, and then transfer the concentrated solution to a centrifuge tube and dry it under a stream of nitrogen gas. Add 100 µL of hexamethyldisilazane (HMDS), 10 µL of pyridine, and 50 µL of the derivatization reagent BSTFA (containing 1% TMCS). Keep the mixture at 80 °C for 45 min with shaking every 5 min. After derivatization, filter the solution through a 0.22 µm nylon organic membrane filter for GC-MS analysis [42].

An Agilent 7890B/5977B gas chromatograph–mass spectrometer (Agilent, Santa Clara, CA, USA) was applied for GC-MS analysis. A PE-5MS capillary column was used for testing, with helium as carrier gas. After bringing the column temperature up to 50 °C for 5 min, it was increased to 280 °C at a rate of 10 °C per minute with a holding duration of 5 min. The temperatures of the transmission line and ion source were 200 °C and 250 °C, respectively. In full scan mode, electron ionization mass spectra were recorded in the range of 30–550 (*m*/*z*), with a solvent delay time of 4.0 min and an injection volume of 1 μL. The retention time (RT) of the compounds was compared with that of real compounds and data from the literature in order to identify them using the NIST database [72].

### 2.8. Co-Cultured Consortium Establishment

The strain BE17^T^ was co-cultured with three wood-decaying fungi (*Trametes versicolor*, *Gloeophyllum trabeum*, and *Schizophyllum commune*) on PDA-NA composite medium plates (composed of 200 g peeled and diced potato, 20 g glucose, 10 g tryptone, 3 g beef extract, 5 g NaCl, 20 g agar, and 1000 mL distilled water, with pH adjusted to 6.0) to assess their compatibility. Strain BE17^T^ and the fungi were activated separately in LB and PDA medium, respectively. Using 6 mm diameter punchers, fungi were punched from the activated fungal plates at equal distances from the center and transferred to the center of the PDA-NA plates. Then, 2 μL of bacterial culture was inoculated using a sterile pipette tip about 2 cm from the fungal disc on the composite medium plates. The plates were incubated at 28 °C, and the growth of the strains was monitored periodically.

Monoculture of bacteria (strain BE17^T^), monocultures of fungi (*Trametes versicolor*, *Gloeophyllum trabeum*, and *Schizophyllum commune*), and compatible bacterial–fungal co-cultures were inoculated into lignin degradation medium. The pH of the medium was adjusted to 6.0 and supplemented with 25 mM MES buffer to maintain pH stability during long-term incubation. In this study, a 1% (*v*/*v*) bacterial inoculum was defined as 1 mL of bacterial seed culture per 100 mL of liquid medium. A 1% fungal inoculum was defined as one 6 mm diameter agar plug containing actively growing mycelium per 100 mL of liquid medium. For co-culture experiments, bacterial and fungal inocula were each set at 2% (*v*/*v*) with a 1:1 ratio, maintaining a total inoculum volume of 4% (*v*/*v*). The cultures were incubated at 30 °C on a rotary shaker at 150 rpm for 15 days. Samples were taken every 3 days to measure microbial growth (OD_600_), lignin degradation rate, and lignin-degrading enzyme activities. The different performances among the bacterial monoculture, fungal monocultures, and co-cultured systems during lignin degradation were analyzed. All experiments were performed in triplicate (n = 3), and results are expressed as mean ± standard deviation.

### 2.9. Analysis of Enzymatic Activity

The cell-free supernatant obtained by centrifugation of 1 mL of monoculture or co-cultured samples at 12,000 rpm for 5 min was used as an enzyme source to assess the activity of laccase and manganese peroxidase. The activity of the laccase was evaluated by observing the oxidation of ABTS at 420 nm (ε_420_ = 36,000 mol^−1^ cm^−1^) [73]. One unit (U) of laccase activity was defined as the quantity of enzyme needed to oxidize 1 nmol of ABTS per minute per liter of liquid sample at 30 °C and pH 4.5. Manganese peroxidase activity was determined by measuring the oxidation of 2,6-dimethoxyphenol (2,6-DMP) to coerulignone, monitored at 469 nm (ε_469_ = 49,600 mol^−1^ cm^−1^) [74]. The 1 mL reaction system consisted of 0.1 mL MnSO_4_ solution (10 mmol/L), 0.1 mL guaiacol solution (10 mmol/L), 0.6 mL succinate–sodium succinate buffer (pH 4.5, 50 mmol/L), and 0.1 mL fermentation supernatant. The reaction was started by adding 0.1 mL of H_2_O_2_ solution (10 mmol/L), and then, the mixture was incubated at 30 °C for 3 min. The change in absorbance at 465 nm was measured. Manganese peroxidase (MnP) activity was expressed in units (U), where one unit corresponds to the amount of enzyme required to oxidize 1 nmol of guaiacol per minute per liter of liquid sample under conditions of 30 °C and pH 4.5.

(1) $$\mathrm{Enzyme}\;\mathrm{activity}\;(U)=\frac{∆OD}{\varepsilon \times b\times ∆t}\times \frac{V\;total}{V\;enzyme}\times 10^{9}$$

Δ*_OD_*: Variation in absorbance during the reaction period

ε: Molar extinction coefficient (M^−1^·cm^−1^), ABTS: ε_420_ = 36,000 L/(mol·cm); guaiacol: ε_465_ = 12,100 L/(mol·cm)

*b*: Path length of the cuvette (cm)

Δ*t*: Reaction time (min)

*V_total_*: Total volume of the reaction system (mL)

*V_enzyme_*: Volume of enzyme sample used in the reaction (mL)

### 2.10. Optimization of Culture Conditions for Lignin Degradation of the Consortium

To investigate the effects of pH, temperature, and bacterial inoculation time on lignin degradation by the co-cultured system, a single-factor experimental design was employed. The pH of the medium was set to 4.0, 5.0, 6.0, 7.0, and 8.0 (with temperature fixed at 30 °C and bacterial inoculation on day 6); incubation temperatures were set to 20, 25, 30, 35, and 40 °C (with pH fixed at 6.0 and bacterial inoculation on day 6); and bacterial inoculation times were set to day 0, 3, 6, 9, and 12 (with pH fixed at 6.0 and temperature at 30 °C). In all experiments, both bacteria and fungi were inoculated at 2% (*v*/*v*) with a 1:1 ratio, and the cultures were incubated for 15 days at 150 rpm. Samples were collected every 3 days to measure microbial growth (OD_600_), lignin degradation rate, and ligninolytic enzyme activities. Based on the lignin degradation rate as the evaluation index, the optimal condition for each factor was identified. Under these optimal conditions, the co-cultured system was inoculated into lignin degradation medium to verify its degradation performance by determining microbial growth, lignin degradation rate, and enzyme activities. Meanwhile, SEM and FTIR were employed to investigate the morphology and chemical structure of alkaline lignin treated by the co-cultured system.

### 2.11. Data Analyses

All experimental data are expressed as mean ± standard deviation (SD) from three independent replicates. One-way analysis of variance (ANOVA) was performed using IBM SPSS Statistics 27. When significant differences were detected (*p* < 0.05), Tukey’s honestly significant difference (HSD) post hoc test was applied for multiple comparisons among group means. Different lowercase letters indicate statistically significant differences (*p* < 0.05) between groups.

## 3. Results and Discussion

### 3.1. Isolation, Identification, and Phylogenetic Analysis of Strain BE17^T^

**Figure 2:**
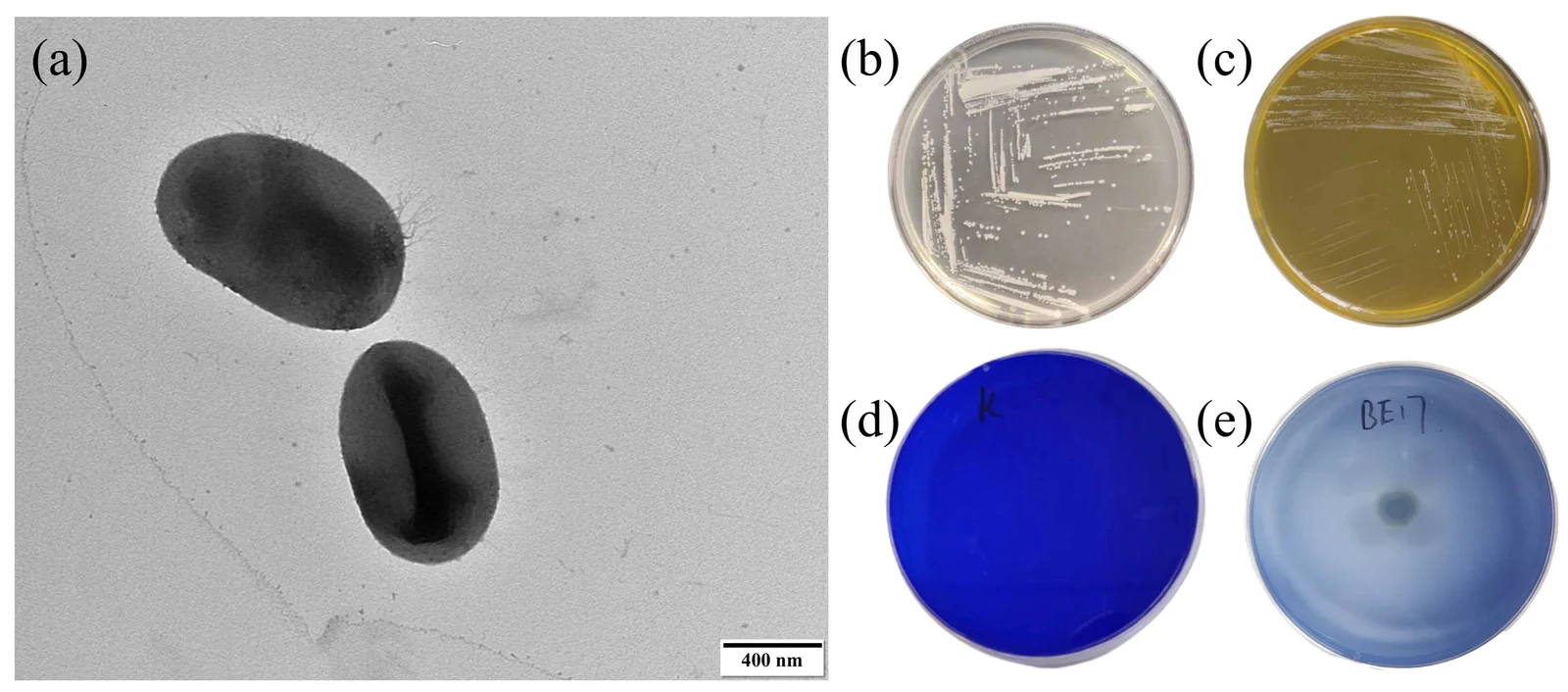
Cell morphology of strain BE17^T^ (**a**, TEM), colony morphology (**b**), utilization of alkaline lignin as the sole carbon source (**c**), and aniline blue plates before (**d**) and after (**e**) decolorization.

The colony labeled as BE17^T^ was selected and preserved at the China Marine Culture Collection Center (MCCC 1K08845) and the Japan Collection of Microorganisms (JCM 36600). The cell morphology of strain BE17^T^ is characterized as short rod-shaped, measuring 0.6–0.7 × 0.9–1.1 μm. The cells possess fimbriae but lack flagella and motility, and they are Gram negative. The colonies are milky white, circular, glossy, with a smooth surface, raised center, regular edges, and are easy to pick up. Strain BE17^T^ can grow on plates with alkaline lignin as the sole carbon source and can decolorize aniline blue plates (Figure 2).

**Figure 3:**
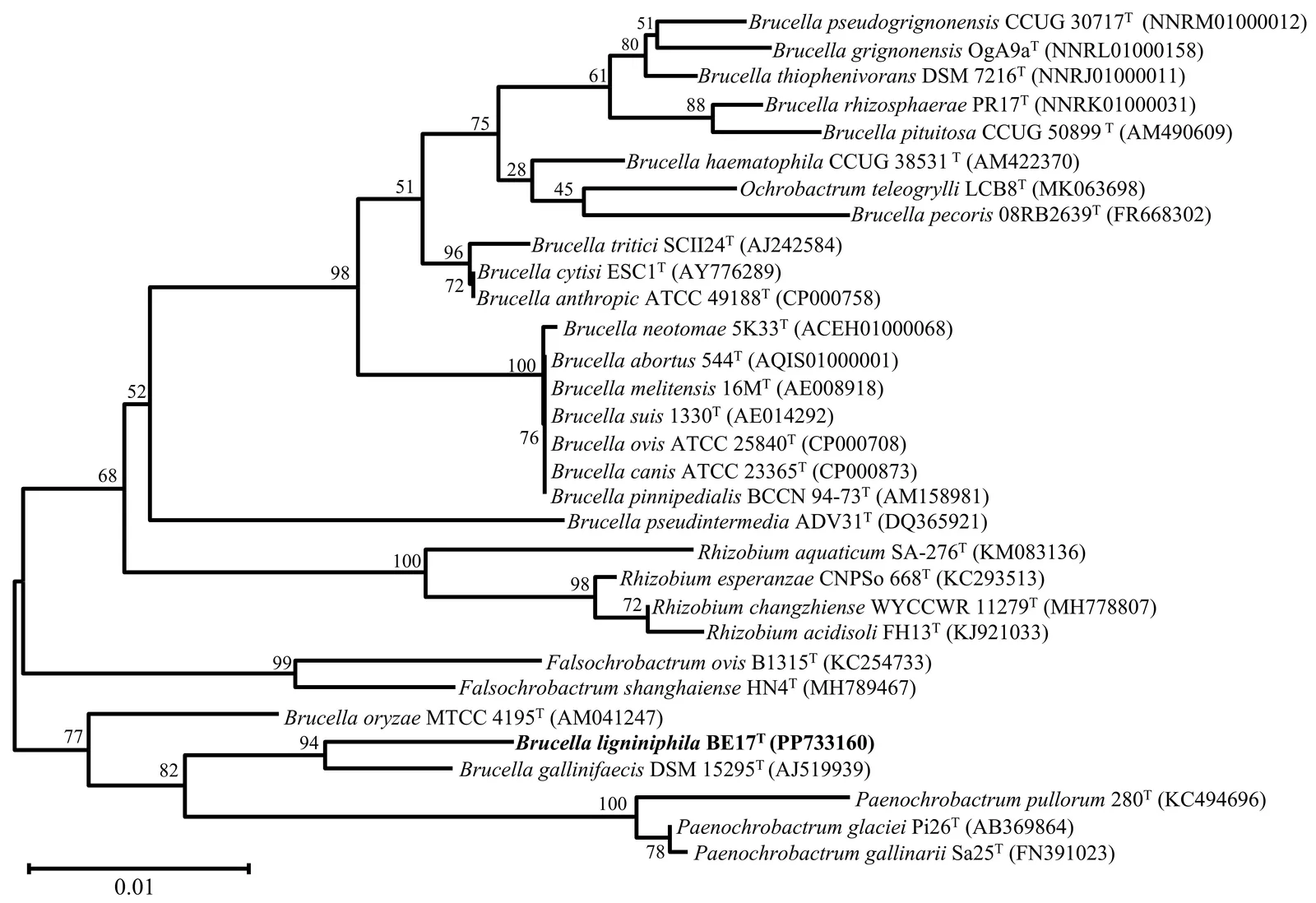
The neighbor-joining phylogenetic tree based on 16S rRNA gene sequences. Bootstrap values based on 1000 replicates are listed as percentages at branching points. Bar, 0.01 substitutions per nucleotide position.

Sequencing results revealed that the 16S rRNA gene sequence of strain BE17^T^ was 1406 bp in length. The complete sequence has been submitted to the GenBank database, and the accession number PP733160 has been obtained. The 16S rRNA gene sequence of strain BE17^T^ showed the highest similarity with *B. gallinifaecis* DSM 15295^T^ (98.58%), followed by *Paenochrobactrum glaciei* Pi26^T^ (96.94%) and *Brucella oryzae* MTCC 4195^T^ (96.80%). The 16S rRNA gene sequences of these closely related strains were downloaded, and an NJ tree was constructed, as shown in Figure 3. The results indicated that strain BE17^T^ is most closely related to *B. gallinifaecis* DSM 15295^T^, which also had the highest 16S rRNA gene similarity. Both phylogenetic and similarity analysis confirmed that *B. gallinifaecis* DSM 15295^T^ is the closest relative of strain BE17^T^. Phylogenomic analysis revealed the similar results (Figure S1).

### 3.2. Analysis of Physiological and Biochemical Characteristics

By evaluating the growth-limiting factors (temperature, salinity, and pH) of strain BE17^T^, it was found that the strain exhibited strong adaptability and robust growth under a wide range of environmental conditions. The temperature, salinity, and pH ranges for growth were 4–40 °C, 0–7%, and 5.0–9.0, respectively, with optimal growth observed at 28 °C, 1% salinity, and pH 8.0, respectively. Physiological and biochemical characterization showed that strain BE17^T^ tested positive for oxidase, catalase, hydrogen sulfide (H_2_S) production, nitrate reduction, and nitrite reduction. It exhibited weakly positive results for the hydrolysis of tween 60 and esculin, but negative for tween 20, tween 40, tween 80, starch, carboxymethyl cellulose (CMC), tyrosine, and casein. Both methyl red and Voges–Proskauer (V-P) tests yielded negative results. Antibiotic susceptibility testing revealed that strain BE17^T^ was sensitive to tetracycline, novobiocin, chloramphenicol, streptomycin, and neomycin but resistant to amoxicillin, clindamycin, penicillin, erythromycin, vancomycin, polymyxin B, and nystatin. Strain BE17^T^ was capable of producing acid from various substrates, including glycogen, _L_-arabinose, _D_-xylose, _D_-melezitose, methyl-β-_D_-xylopyranoside, _D_-galactose, _D_-glucose, _D_-fructose, _D_-mannose, mannitol, sorbitol, _D_-cellobiose, xylitol, _D_-lyxose, _D_-tagatose, _D_-fucose, _L_-fucose, _D_-arabitol, and _L_-arabitol. It could utilize _D_-fructose, _D_-galactose, _D_-fucose, _L_-fucose, _D_-arabitol, _D_-serine, _L_-aspartic acid, _D_-galacturonic acid, _L_-galactono-1,4-lactone, _D_-glucuronic acid, glucuronamide, methyl pyruvate, α-ketoglutaric acid, _D_-malic acid, _L_-malic acid, and acetic acid as carbon sources. Table 1 summarizes the detailed phenotypic characteristics. The results indicated that many traits could differentiate strain BE17^T^ from the reference strain *B. gallinifaecis* DSM 15295^T^. For example, strain BE17^T^ was negative for the methyl red test and positive for nitrite reduction, whereas the reference strain was weakly positive and negative, respectively. In addition, strain BE17^T^ was resistant to penicillin and erythromycin, while the reference strain was not. Furthermore, strain BE17^T^ could produce acid from multiple substrates such as _L_-arabinose, _D_-mannose, and _D_-sorbitol, which were not utilized by the reference strain *B. gallinifaecis* DSM 15295^T^.

### 3.3. Chemotaxonomic Characterization Analysis

The results of chemotaxonomic analysis indicated that the predominant respiratory quinone in strain BE17^T^ is Q10, which is consistent with the descriptions of other species within the genus *Brucella*. This finding further confirms the taxonomic placement of strain BE17^T^ within the genus *Brucella*. The polar lipid profile of strain BE17^T^ comprises phosphatidylethanolamine (PE), phosphatidylmonomethylethanolamine (PEM), phosphatidylglycerol (PG), phosphatidylcholine (PC), three unidentified phospholipids (PL_1-3_), two unidentified aminolipids (AL_1-2_), two unidentified glycolipids (GL_1-2_), and several other unidentified polar lipids (L_1-2, 5-6_). The polar lipid profile of strain *B. gallinifaecis* DSM 15295^T^ is similar to that of strain BE17^T^, but it lacks one phospholipid (PL_3_) and includes two additional unidentified polar lipids (L_3-4_) (Figure S2). The fatty acid analysis revealed that the predominant fatty acid in strain BE17^T^ is summed feature 8 (C_18:1_ *ω*7*c*; 60.20%), followed by C_19:0_ cyclo *ω*8*c* (12.94%), C_18:1_ 2OH (12.39%), and C_16:0_ (3.63%). In contrast, the reference strain *B. gallinifaecis* DSM 15295^T^ contents summed feature 8 (51.14%), C_19:0_ cyclo *ω*8*c* (25.63%), C_16:0_ (7.73%), and C_18:1_ 2OH (4.61%). Although the main types of fatty acids are the same between strain BE17^T^ and *B. gallinifaecis* DSM 15295^T^, their relative abundances show significant differences (Table 2).

### 3.4. Genomic Characterization Analysis

The genome sequence information of strain BE17^T^ was obtained using Nanopore and Illumina platforms. The genome completeness was assessed to be ≥ 95% (99.13%), with contamination ≤ 5% (0%), making it an ideal reference strain for further analysis. The sequencing results revealed that the genome size of strain BE17^T^ is 3,888,332 bp, consisting of 2 circular chromosomes without any plasmids. The average G+C content of the genome is 54.86%. There is a total of 3697 protein-coding genes, with a cumulative length of 3,407,808 bp, accounting for 87.64% of the total genome length (Table 3). The whole genome sequence is available in the GenBank database with the accession numbers CP154467 and CP154468.

**Figure 4:**
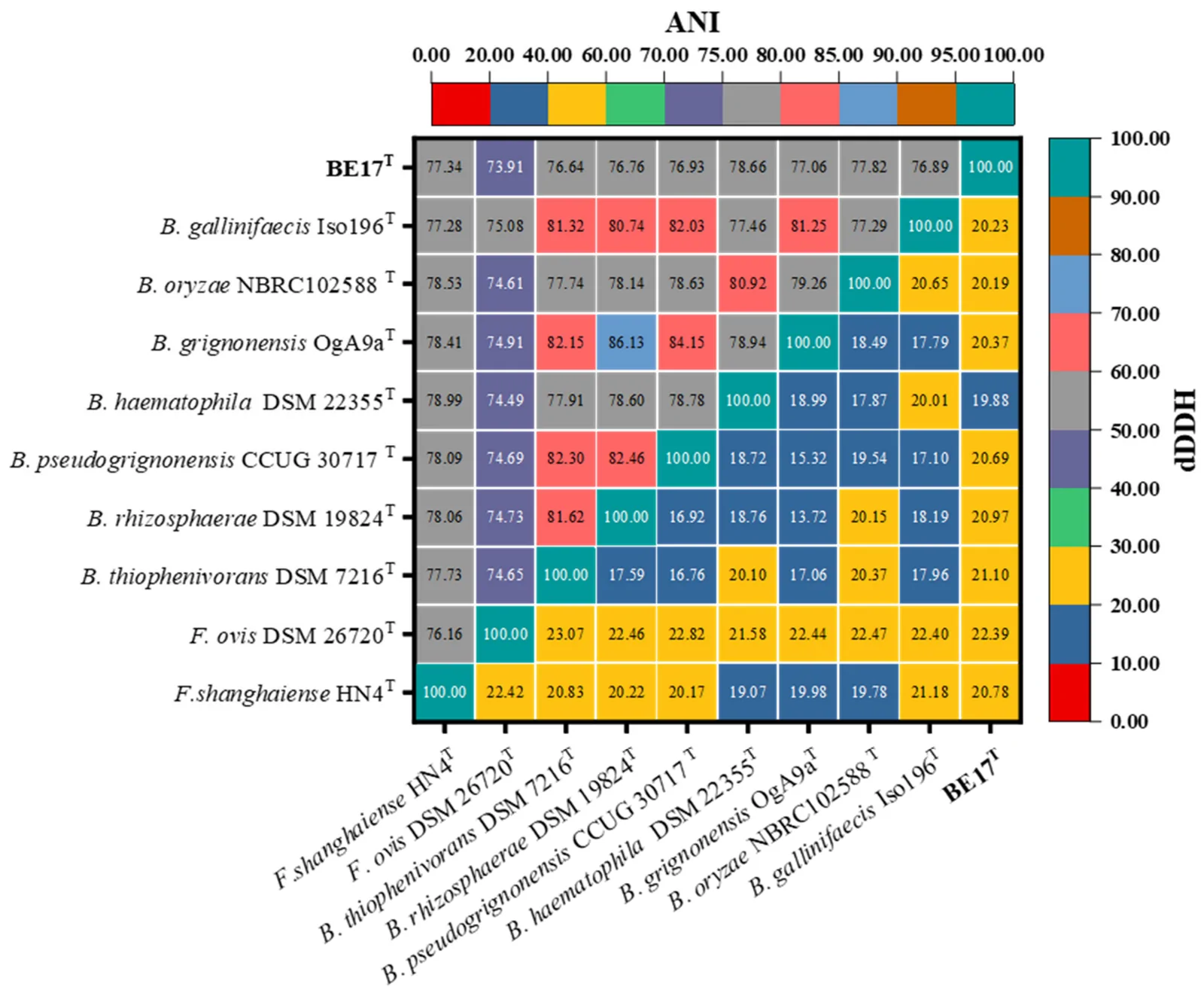
ANI and dDDH values between strain BE17^T^ and relative strains.

According to calculations using OAT software v0.93.1, strain BE17^T^ exhibits an average nucleotide identity (ANI) of 76.89% with strain *B. gallinifaecis* DSM 15295^T^, and ANI values with other similar strains also range between 70% and 80%. These values are notably lower than the ANI threshold (94–96%) typically used for defining new species [75]. Additionally, digital DNA–DNA hybridization (dDDH) values, another criterion for species delineation, indicate that strain BE17^T^ shares approximately 20% dDDH with similar strains. This finding similarly falls well below the conventional 70% threshold utilized in prokaryotic species descriptions (Figure 4) [76]. Based on the phenotypic, chemotaxonomic, and genotypic characteristics of strain BE17^T^, this study proposes that the strain represents a new species within the genus *Brucella*. Therefore, we suggest the establishment of a new species within the *Brucella* genus, named *Brucella ligniniphila*, with the type strain *Brucella ligniniphila* BE17^T^, and the strain preservation numbers MCCC 1K08845^T^ and JCM 36600^T^.

### 3.5. Analysis of Lignin Degradation Performance of Strain BE17^T^

**Figure 5:**
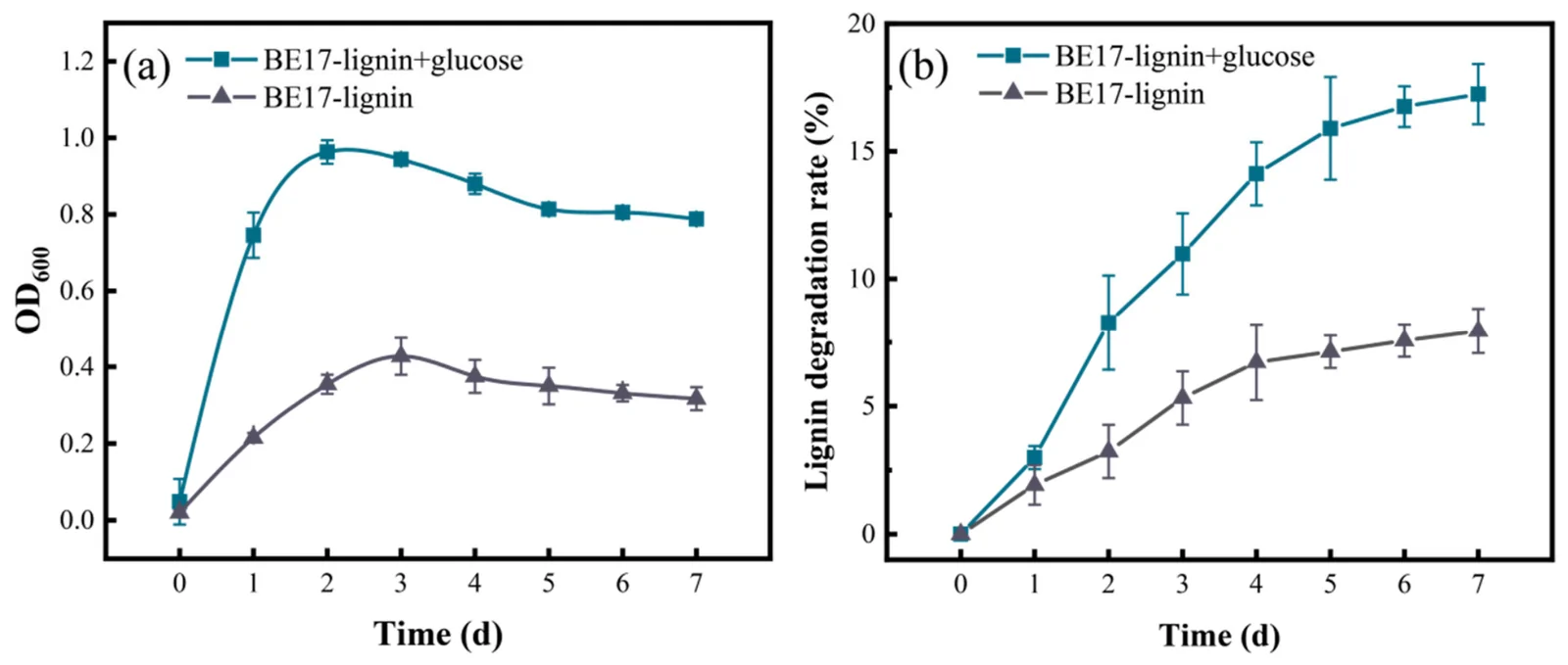
Growth (**a**) and lignin degradation (**b**) curves of strain BE17^T^.

Strain BE17^T^ was inoculated into lignin degradation medium with and without the addition of glucose as a supplementary carbon source and cultured for 7 days. During the exponential growth phase (days 0–2), the bacterial cell concentration increased exponentially, with cells actively dividing and exhibiting high metabolic activity (Figure 5a). At this stage, cells absorb external nutrients such as carbon, nitrogen, and trace elements to support their growth and metabolism. The significant increase in lignin degradation rate within the first three days also confirms that strain BE17^T^ actively degrades lignin during this period through various metabolic processes (Figure 5b). Upon reaching the stationary phase, the metabolic rate decreased and reached a dynamic balance. By day 7, the lignin degradation rate reached 7.9% with lignin as the sole carbon source and 17.2% with glucose supplementation. The results indicated that the addition of glucose significantly enhanced both cell growth and lignin degradation efficiency compared with using lignin as the sole carbon source. Furthermore, in the presence of glucose, cell metabolism entered the stationary phase more rapidly. This is because lignin, as a complex high-molecular-weight aromatic polymer, is difficult for bacteria to efficiently degrade and utilize in the early stages of growth, thus limiting the energy available for metabolism [77]. Previous studies also have shown that supplementation with simple carbon sources such as glucose and xylose can meet the nutritional needs for bacterial proliferation more effectively, enabling rapid metabolism and enzyme production, thereby facilitating lignin degradation [77,78,79].

**Figure 6:**
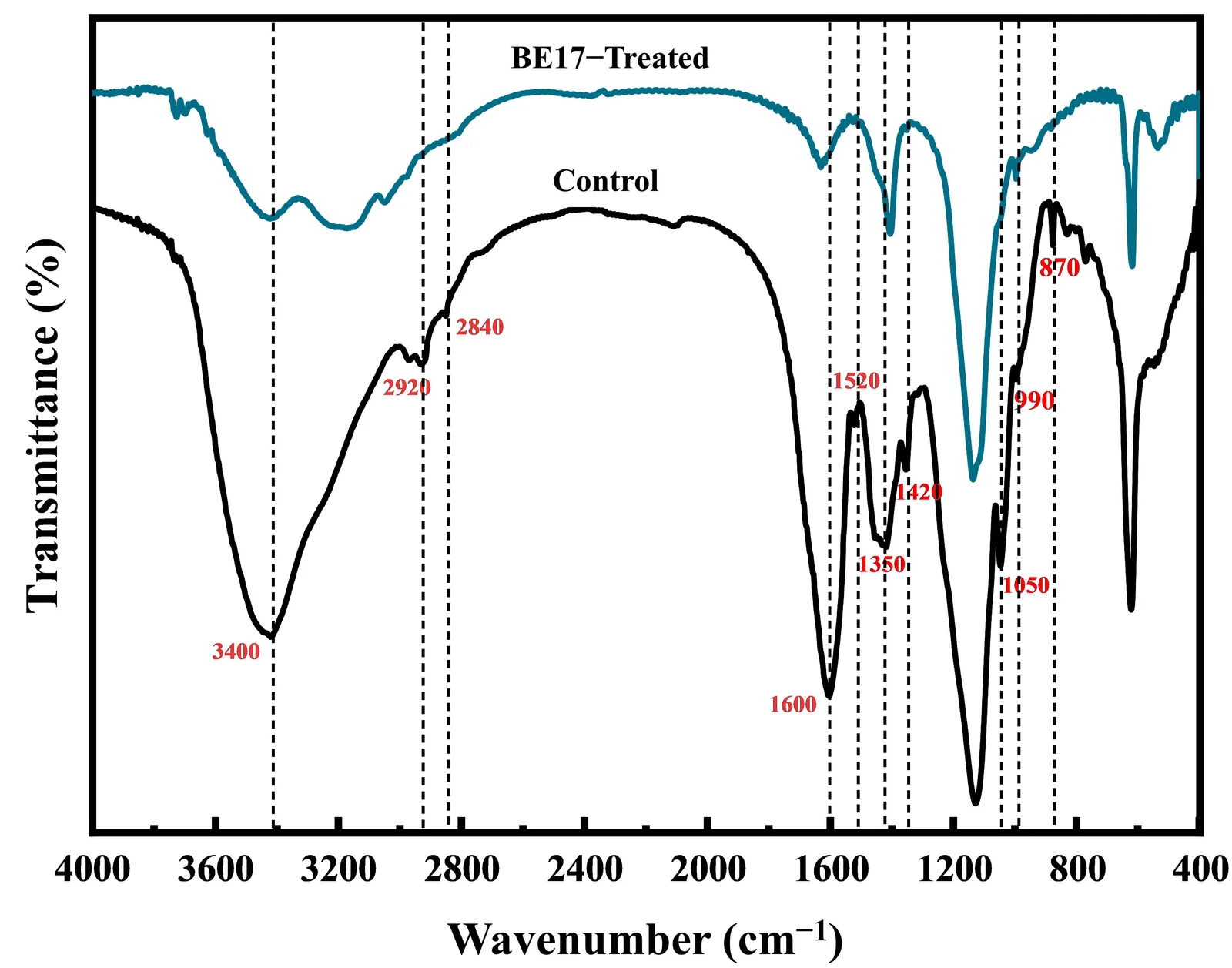
Changes in the chemical structure of lignin degraded by strain BE17^T^ revealed by FTIR.

To investigate the chemical structural changes of lignin before and after degradation by strain BE17^T^, Fourier transform infrared spectroscopy (FT-IR) was performed. As shown in Figure 6, the FT-IR spectrum of the treated group exhibited significant changes compared with the control group. The intensity of the hydroxyl (–OH) absorption peak around 3400 cm^−1^ was markedly reduced, indicating that phenolic hydroxyl groups were oxidized or involved in condensation reactions, possibly forming quinones or ether compounds [80]. The disappearance of C–H stretching vibration peaks around 2920 cm^−1^ and 2840 cm^−1^ suggests cleavage or oxidation of lignin side chains such as CH_2_, CH_3_, and CH_3_O groups, potentially leading to the formation of carboxylic acids or ketones [81,82,83]. The aromatic C=C skeletal vibration peak at 1600 cm^−1^ and the C–C vibration peak at 1520 cm^−1^ were significantly weakened or disappeared, directly indicating disruption of the aromatic ring structure, possibly through demethylation or ring-opening reactions that produce small-molecule phenolic acids [84,85]. The methoxy C–H bending vibration peak at 1420 cm^−1^ showed a slight shift, further supporting the demethylation and conversion of methoxy (–OCH_3_) to hydroxyl (–OH) groups [86]. The disappearance of the C–O stretching vibration peak at 1050 cm^−1^ indicates cleavage of the β–O–4 ether bonds in lignin, which is key evidence for lignin depolymerization [82]. The enhanced absorption peak at 990 cm^−1^ may correspond to the formation of monophenolic compounds, while the disappearance of the out-of-plane bending vibration peak of the aromatic ring at 870 cm^−1^ may suggest a reduction in G/S-type aromatic units [85,87]. The FTIR spectra of the treated samples displayed considerable differences from those of the control samples, indicating that strain BE17^T^ had extensively degraded the lignin structure. Specifically, strain BE17^T^ tends to initiate lignin depolymerization by attacking β–O–4 ether linkages, oxidizing aliphatic side chains, and disrupting aromatic ring structures, thereby generating intermediate metabolites through demethylation and ring-opening reactions.

**Figure 7:**
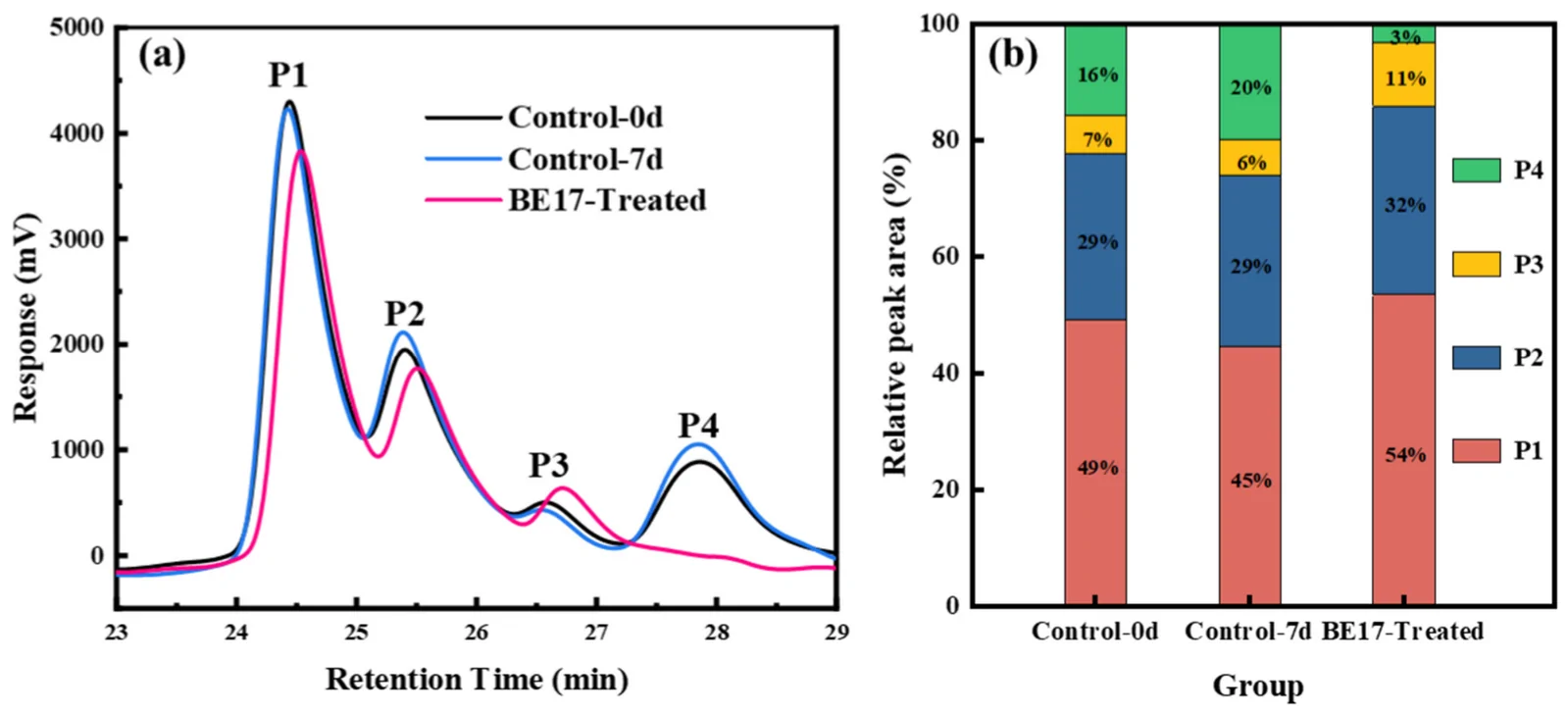
Changes in molecular weight of lignin before and after degradation by strain BE17^T^ revealed by GPC. (**a**) GPC chromatograms of molecular weight distribution; (**b**) Stacking diagram of molecular weight distribution.

Gel permeation chromatography (GPC) was used to analyze the molecular weight distribution characteristics of lignin before and after degradation by strain BE17^T^. The samples labeled Control-0d, Control-7d, and BE17-treated correspond to lignin at day 0 and day 7 of the uninoculated control group and lignin at day 7 after BE17^T^ inoculation, respectively. As shown in Figure 7a, the elution times of the control samples at day 0 and day 7 were nearly identical, indicating no significant change in molecular weight and suggesting that lignin maintained structural stability and did not undergo self-degradation during the 7-day shaking incubation. In contrast, the elution time of the GPC chromatogram for the BE17-treated sample shifted toward longer retention times, indicating a decrease in molecular weight. Additionally, changes in the peak areas at positions P1, P2, and P3 were relatively minor between the control and treated groups, whereas a significant reduction was observed at P4 (Figure 7b), suggesting that strain BE17^T^ has limited ability to directly utilize high-molecular-weight lignin and that low-molecular-weight lignin is more readily degraded and transformed.

### 3.6. Lignin Degradation Mechanism Analysis of Strain BE17^T^

#### 3.6.1. Aniline Blue Decolorization Analysis

**Figure 8:**
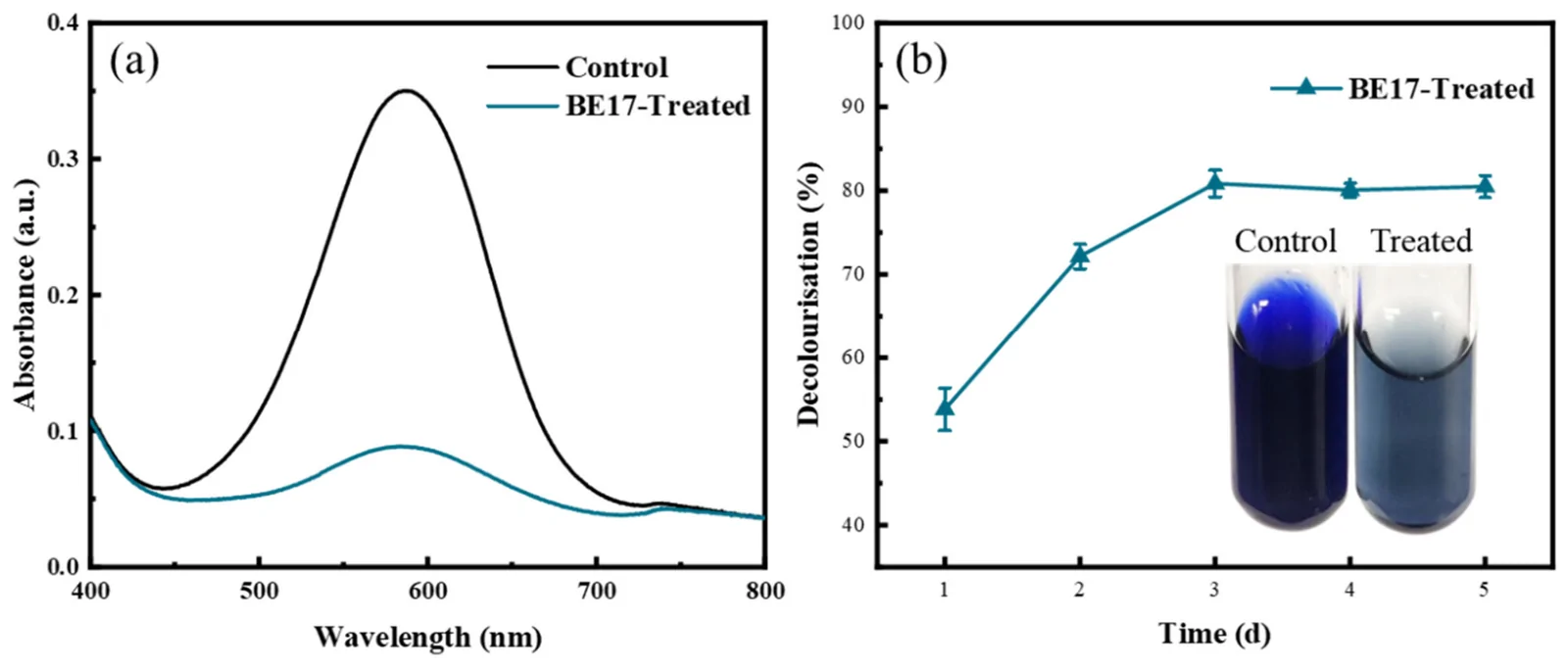
Aniline blue decolorization of strain BE17^T^. (**a**) UV–vis spectra of aniline blue before and after degradation by strain BE17^T^; (**b**) Decolorization rate of aniline blue by strain BE17^T^.

Strain BE17^T^ was inoculated into LB liquid medium containing 1% aniline blue and cultured for 5 days. Visual observation showed that BE17^T^ exhibited strong decolorization ability toward aniline blue. After centrifugation of the fermentation broth, the supernatant was appropriately diluted and scanned using a UV-vis spectrophotometer across the 400–800 nm range. The results showed a distinct absorption peak at 590 nm for aniline blue, which was significantly weakened after treatment with BE17^T^ (Figure 8a), indicating degradation of the aniline blue molecules. The decolorization rate was calculated by measuring the change in absorbance at 590 nm. The results showed a significant increase in decolorization within the first 1–3 days, reaching a maximum rate of 80.9% on day 3, after which no further increase was observed (Figure 8b). The notable decolorization capacity of strain BE17^T^ is likely due to the secretion of oxidative enzymes, including dye-decolorizing peroxidases. These enzymes catalyze redox reactions that break the azo bonds in aniline blue molecules, leading to the formation of colorless or faint-colored small molecular compounds [88]. In particular, dye-decolorizing peroxidases (DyPs) are known not only for their efficient degradation of azo dyes but also for their activity against various lignin-derived aromatic compounds [89]. As key enzymes in lignin biodegradation, DyPs possess broad substrate specificity capable of oxidizing both phenolic and non-phenolic lignin analogs. Their reactions often involve cleavage of the Cα-Cβ bond [90] or oxidative polymerization of phenol groups [91]. Ahmad et al. [18] identified a DyP-type peroxidase (DyPB) from *Rhodococcus jostii* RHA1 that, in the presence of Mn^2+^, demonstrated a 5–23-fold increase in catalytic efficiency. Moreover, DyPB was able to convert β-aryl ether-type lignin model compounds into vanillin, suggesting its capacity to cleave the Cα-Cβ bond on the aromatic side chain. Therefore, the performance of strain BE17^T^ in the aniline blue decolorization experiment not only confirms its strong dye degradation ability but also suggests its broad application potential in lignin biodegradation and environmental remediation.

#### 3.6.2. Lignin-Degrading Enzyme Coding Genes in Strain BE17^T^

Currently, the most widely reported bacterial enzymes involved in lignin degradation include multicopper oxidase laccase (Lac), lignin peroxidase (LiP), manganese peroxidase (MnP), versatile peroxidase (VP) and dye-decolorizing peroxidase (DyP) [92]. In addition to these major lignin-degrading enzymes, many auxiliary enzymes also participate in lignin degradation metabolism, such as oxidases, glutathione-dependent β-etherases, catalases, transferases, dehydrogenases, decarboxylases, monooxygenases, dioxygenases, and superoxide dismutases [93]. These enzymes indirectly contribute to various metabolic processes during bacterial lignin degradation [93]. According to genome annotation results and the relevant literature on the functions of lignin-degrading enzyme genes, a total of 93 lignin degradation-related enzyme genes were identified, and some are listed in Table 4. Two multicopper oxidase gene sequences (gene 3579 and gene 1581) were observed in the genome of strain BE17^T^. In addition, a dye-decolorizing peroxidase gene (gene 2896) was also identified, consistent with experimental results showing that strain BE17^T^ possesses dye decolorization capability, confirming its potential to secrete DyP-type peroxidases. However, no genes encoding Lac, MnP or LiP were found in the genome. Although strain BE17^T^ lacks a canonical laccase gene, it harbors a DyP-type peroxidase, which is known to oxidize a broad range of substrates including both phenolic and non-phenolic lignin model compounds. This enzyme may play a central role in the oxidative cleavage of lignin, potentially compensating for the absence of laccase. The two multicopper oxidases identified in the genome may also contribute to lignin modification, though their substrate specificity and requirement for mediators remain to be characterized. The results of signal peptide analysis revealed that two multicopper oxidases (gene 3579, gene 1581) and the DyP-type peroxidase (gene 2896) contain TAT (twin-arginine translocation) signal peptides, with high prediction probabilities of 0.957, 0.997, and 0.995 for the two oxidases and the DyP, respectively. The TAT pathway is specifically responsible for the secretion of fully folded proteins, particularly those that require cofactor incorporation, such as copper-containing multicopper oxidases, prior to transport [94]. The identification of TAT signal peptides suggests that BE17^T^ employs the TAT secretion system to transport these ligninolytic enzymes across the cytoplasmic membrane, a feature that has been documented in other Gram-negative lignin-degrading bacteria such as *Pseudomonas aeruginosa* [95].

Other genes related to lignin degradation metabolism were also identified, including catechol 2,3-dioxygenase (gene 0857), aromatic ring-hydroxylating dioxygenase (gene 2754), aromatic ring-opening dioxygenase (gene 3450), prephenate dehydratase (gene 2074), phenylacetic acid degradation protein (gene 1102), catalase (gene 2549), coenzyme A transferase (gene 0714), glutathione S-transferase (gene 0455), methyltransferase (gene 0139), and superoxide dismutase (gene 0327). Notably, strain BE17^T^ also carries a full phenylacetate (paa) pathway (paaG, paaC, paaH, paaF, paaK, paaN, paaY), which was able to convert aromatic acids (via CoA activation) into ring-fission products that yield acetyl-CoA and succinyl-CoA [96]. The presence of the complete paa gene cluster in strain BE17^T^ indicates that the strain has evolved a specialized mechanism for processing phenylacetic acid and related compounds. This pathway is particularly relevant to lignin degradation because phenylacetic acid can be derived from the breakdown of lignin’s phenylpropanoid units, making it a key intermediate in lignin valorization.

We acknowledge that while genomic analysis reveals the genetic potential for lignin degradation, proteomic analysis of the extracellular secretome would be required to confirm that these enzymes are actually produced and secreted during lignin degradation. Such analyses, including mass spectrometry-based quantitative proteomics of the culture supernatant, will be essential to validate the involvement of specific enzymes in the degradation process and will be addressed in our future studies.

#### 3.6.3. Analysis of Lignin Degradation Products of Strain BE17^T^

Compounds in the fermentation broth were extracted using ethyl acetate, and the lignin degradation products of strain BE17^T^ were analyzed by GC-MS. A total of 27 intermediates from the degradation process were identified, which primarily consisted of 6 distinct classes of compounds. Specifically, there were 15 organic acids: 2-methylbenzoic acid (**3**), hexanoic acid (**4**), acetoacetic acid (**5**), 3-ketovaleric acid (**6**), 2-5methylacetoacetic acid (**7**), hexadecanoic acid (**11**), hexanedioic acid (**13**), 1,2-benzenedicarboxylic acid (**14**), vanillic acid (**15**), 3-methoxy-4-hydroxymandelic acid (**16**), p-coumaric acid (**17**), α-hydroxycinnamic acid (**20**), 2-hydroxy-5-methylbenzoic acid (**21**), 2-tolylacetic acid (**22**), and 1,3-benzenedicarboxylic acid (**25**); four esters: ethyl malonate (**8**), dibutyl phthalate (**18**), ethyl ester (**19**), and bis(2-ethylhexyl) phthalate (**24**); two phenols: phenol (**9**) and 3,5-dimethylphenol (**26**); three aldehydes and ketones: p-ethylbenzaldehyde (**2**), vanillin (**10**), and 2′,6′-dihydroxyacetophenone (**23**); two alcohols: 2-methoxyethanol (**1**) and 4-hydroxybenzyl alcohol (**27**); and one alkene: cetene (**12**). These 27 low-molecular-weight compounds are associated with lignin degradation metabolism. Their serial numbers, retention times, and names are listed in Table 5. It is worth noting that vanillin, vanillic acid, *p*-coumaric acid, dibutyl phthalate, and 1,3-benzenedicarboxylic acid were found in control and treated samples, which may be attributed to the chemical oxidation of lignin due to aeration and agitation [12,85].

Microbial degradation of lignin mainly occurs in two steps: firstly, lignin is depolymerized into low-molecular-weight aromatic oligomers by microbial extracellular enzymes and transported into cells; secondly, these monomers are funneled through metabolic pathways into central carbon metabolism and converted into low-molecular-weight aromatic or other organic compounds [97,98]. The sample inoculated by strain BE17^T^ had significantly more compounds than the control. A total of 12 aromatic compounds were detected only in the inoculated sample: p-ethylbenzaldehyde (**2**), 2-methylbenzoic acid (**3**), phenol (**9**), 1,2-benzenedicarboxylic acid (**14**), 3-methoxy-4-hydroxymandelic acid (**16**), α-hydroxycinnamic acid (**20**), 2-hydroxy-5-methylbenzoic acid (**21**), 2-tolylacetic acid (**22**), 2′,6′-dihydroxyacetophenone (**23**), bis(2-ethylhexyl) phthalate (**24**), 3,5-dimethylphenol (**26**), and 4-hydroxybenzyl alcohol (**27**).

2-Methylbenzoic acid (o-toluic acid) is a substituted benzoic acid (CH_3_–C_6_H_4_–COOH) that can arise during aromatic ring breakdown. In the context of lignin biodegradation, 2-methylbenzoic acid is not one of the classical lignin monomer units, but it has been detected as a minor aromatic breakdown product in several studies. *Serratia* sp. AXJ-M were found to produce or release small amounts of 2-methylbenzoic acid when degrading lignin [99]. A recent study of *Erwinia billingiae* QL-Z3 revealed that under enzyme-driven lignin breakdown, syringyl and guaiacyl units were converted through multi-step reactions into intermediates like 3-methoxyphenylacetic acid, which was then further oxidized to yield 2-methylbenzoic acid [83]. These observations indicate that 2-methylbenzoic acid does occur as a byproduct of microbial lignin breakdown. Moreover, 2-methylbenzoate does not typically persist, as it is rapidly catabolized by ring-cleavage enzymes.

1,2-Benzenedicarboxylic acid (phthalic acid) is an aromatic dicarboxylic acid with a benzene ring substituted by two ortho-carboxyl groups that shares an aromatic structural homology with lignin. It has been detected as a byproduct in several bacterial lignin-degradation studies. *Streptomyces thermocarboxydus* DF3-3 degrades dibutyl phthalate (a common intermediate) to phthalic acid, and then converts that into syringyl units and protocatechuic acid for further degradation [42]. Likewise, *Bacillus ligniniphilus* L1 produced phthalic acid among the aromatic compounds identified by GC-MS after lignin treatment [77]. Even consortia of *Citrobacter* spp. (from pulp waste sludge) degraded alkaline “black liquor” lignin to produce phthalic anhydride (which hydrolyzes to phthalic acid) along with other aromatic fragments [79]. In summary, various lignin-degrading bacteria generate phthalic acid (or its esters/anhydrides) as an intermediate. When microbes do generate phthalic acid, they usually metabolize it further via specialized aromatic pathways. Up to four enzymes are involved in phthalate degradation: phthalate dioxygenase, dihydroxyphthalate dehydrogenases, decarboxylases, and ring-cleaving oxygenases [100]. These enzymes convert phthalic acid into protocatechuate (3,4-dihydroxybenzoate), which is then cleaved by protocatechuate 3,4-dioxygenase (pcaG/pcaH) into β-ketoadipate [42]. In other words, phthalic acid is funneled into the central β-ketoadipate pathway or gentisate pathway for complete mineralization.

α-Hydroxycinnamic acid (2-hydroxycinnamic acid) is not a canonical lignin monomer and occurs only as a trace byproduct, with no dedicated bacterial formation pathway or central metabolic role [101,102]. Similarly, 2′,6′-dihydroxyacetophenone is a rare aromatic ketone reported in only a few studies (e.g., from decayed-wood consortia) and has no established biochemical function in lignin degradation [103,104]. In contrast, 4-hydroxybenzyl alcohol is a more relevant intermediate, originating from the p-hydroxyphenyl (H) units of lignin. Bacteria such as *Pseudomonas putida* sequentially oxidize it to 4-hydroxybenzaldehyde and then to 4-hydroxybenzoate, which enters the protocatechuate branch of central metabolism [105,106]. Thus, 4-hydroxybenzyl alcohol serves as a funneling intermediate rather than a dead-end product. Dibutyl phthalate is a common intermediate of lignin that can be metabolized into 1,2-benzenedicarboxylic acid, which can then be further transformed into compounds such as vanillic acid and protocatechuic acid and subsequently degraded.

Beyond the primary intermediates discussed above, the GC-MS analysis revealed additional compounds that provide insight into the complete degradation pathway. These include various methoxyphenols and catechols, which are key intermediates in the deoxygenation reactions that convert lignin-derived aromatics into stable phenolic compounds. Detection of these compounds indicates that strain BE17^T^ primarily degrades lignin through a pathway that proceeds via methoxyphenols and catechols as intermediates, ultimately generating stable phenols in the aqueous phase. The identification of guaiacol, veratryl alcohol, and 2,6-dimethoxyphenol (2,6-DMP) in previous studies implies the existence of genes, metabolic routes, or enzymatic systems capable of oxidizing both phenolic and non-phenolic lignin monomers. These compounds are particularly significant as they represent different structural classes of lignin degradation products, indicating that strain BE17^T^ possesses a broad substrate specificity for lignin-derived compounds. The integration of all detected intermediates suggests a highly organized degradation pathway that proceeds through multiple parallel and sequential reactions. This complexity indicates that strain BE17^T^ has evolved a sophisticated enzymatic machinery capable of processing the diverse structural units present in lignin, from methoxylated to non-methoxylated aromatic compounds.

#### 3.6.4. Prediction of the Intracellular Lignin Metabolism Pathway of Strain BE17^T^

**Figure 9:**
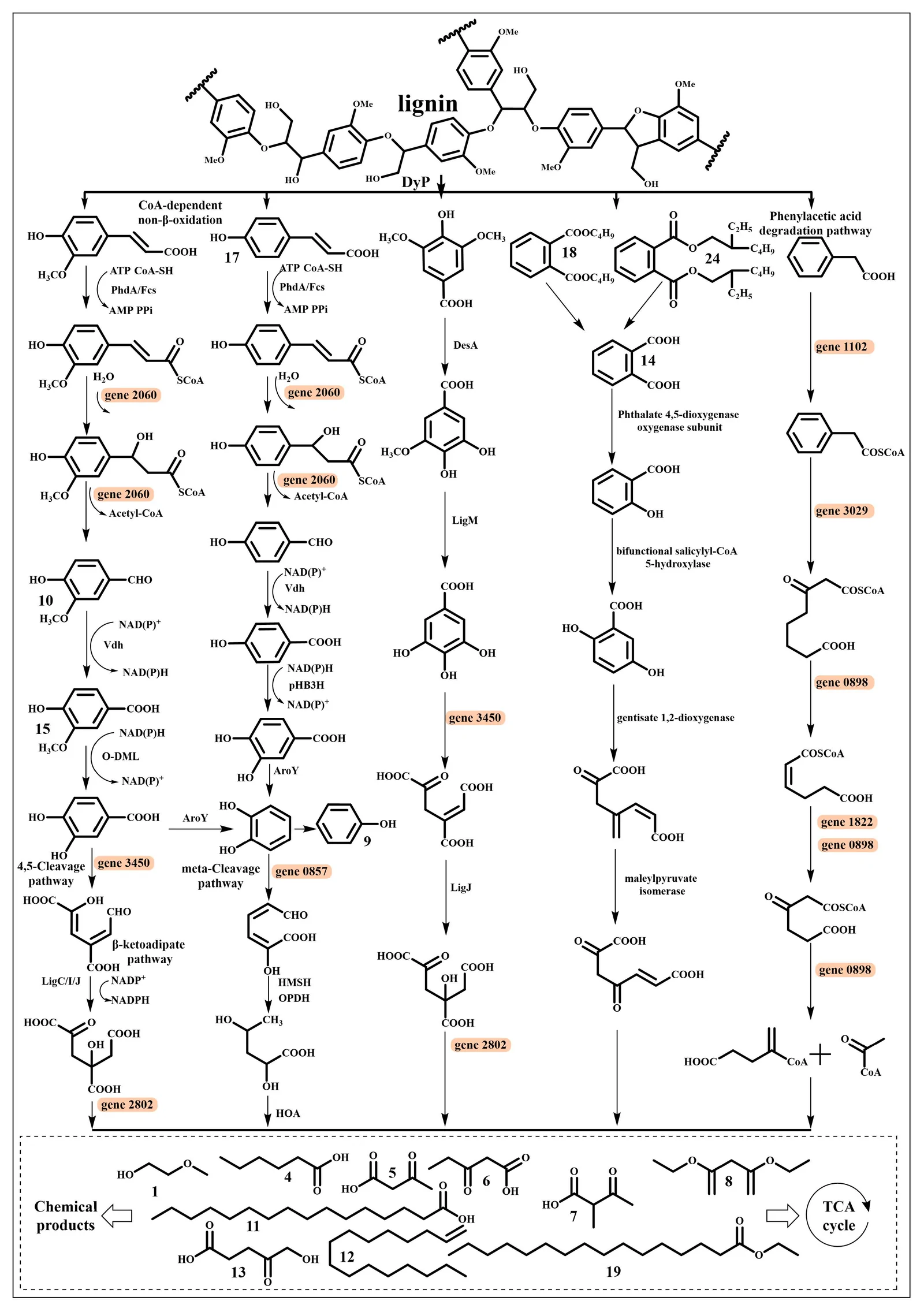
Predicted lignin degradation pathway for strain BE17^T^ based on the genomic and GC-MS analysis obtained from this study.

Many low-molecular-weight aromatic compounds, such as vanillin, vanillic acid, phenol, and phthalic acid, were detected in the GC-MS analysis results. Based on the lignin degradation products of strain BE17^T^ and the analysis of degradation-related enzyme genes, the intracellular lignin metabolism pathway of strain BE17^T^ was predicted (Figure 9). Strain BE17^T^ may first degrade lignin by secreting enzymes such as dye-decolorizing peroxidase, which break the carbon–carbon and ether bonds in lignin, depolymerizing the lignin polymers into lignin-related monomers. These monomers are then transported into the cell by aromatic transport proteins on the cell membrane, such as the major facilitator superfamily (MFS) and tripartite ATP-independent periplasmic (TRAP) transporters. After these small aromatic monomers are transported into the cell, they undergo coenzyme A-dependent non-β-oxidative metabolic pathways (gene 2060), converting lignin monomers like ferulic acid and coumaric acid into intermediates such as protocatechuic acid and catechol. Then, dioxygenases (gene 2060, gene 0857) cleave the aromatic rings, and the β-ketoadipate pathway produces acetylacetic acid and pyruvate, which are central metabolites involved in the tricarboxylic acid (TCA) cycle for cell growth and further conversion into other chemicals. LigK (gene 2802) is a key enzyme gene in the β-ketoadipate pathway, catalyzing the cleavage of 4-carboxy-2-hydroxy-2-oxohexanedioic acid to generate pyruvate and acetoacetate, which then enter the TCA cycle for cellular metabolism. Additionally, genome annotation of enzyme genes revealed that strain BE17^T^ contains multiple enzyme genes involved in phenylacetic acid degradation, comprising paaG (gene 3029), paaC (gene 3190), paaH (gene 1822), paaF (gene 0898), paaK (gene 0094), paaN (gene 3708), and paaY (gene 1102), as listed in Table 4. Those genes suggest that strain BE17^T^ possesses a complete phenylacetic acid metabolic pathway.

The lignin-degradation pathway in strain BE17^T^ shares features with other bacterial systems but also shows distinct traits. Like many soil proteobacteria (e.g., *Pseudomonas*, *Cupriavidus*), BE17^T^ employs dioxygenases and β-ketoadipate-like metabolism for aromatic breakdown [98]. However, several differences stand out. Firstly, the most significant innovation is the presence of the paaG gene and its associated pathway, which enables the strain to process phenylacetic acid derivatives through a unique mechanism involving ring epoxidation and isomerization. This pathway is particularly noteworthy because it can operate under conditions of limited oxygen availability, providing the strain with metabolic flexibility. The pathway also demonstrates remarkable substrate flexibility, as evidenced by the detection of diverse intermediates including p-ethylbenzaldehyde, methoxyphenols, and catechols. This flexibility indicates that strain BE17^T^ has evolved a broad-spectrum approach to lignin degradation that can process both methoxylated and non-methoxylated aromatic compounds. Secondly, the presence of catechol 2,3-dioxygenase (meta-cleavage) and HcaD contrasts with the ortho-cleavage (catechol 1,2-dioxygenase) more common in some lignin catabolizers. This may yield different intermediates (e.g., 2-hydroxymuconic semialdehyde) and explains the ketonic acids detected.

Moreover, *Brucella* spp. are not classic lignin degraders; they are mostly known as mammalian pathogens. Recent metagenomic work noted that *Brucella* (and its relatives *Ochrobactrum*/*Sphingomonas*) do contain lignin-degrading bacteria [102]. Our findings provide one of the first detailed pathways for ligninolysis by a *Brucella* strain, which suggests that even microbes typically associated with hosts can participate in biomass cycling. The novel enzyme combinations in BE17^T^ may inspire engineered microbes or enzyme cocktails for efficient lignocellulose valorization. Moreover, the ability to produce diverse intermediates suggests that it could be engineered to produce specific chemicals of industrial importance, including precursors for pharmaceuticals, polymers, and other specialty chemicals. However, it should be noted that the above pathway is proposed based on the integration of genomic annotation and GC-MS metabolite profiling, and direct validation of each enzymatic step through approaches such as isotopic labeling or in vitro enzyme assays is required in our future research.

### 3.7. Construction of a Bacterial–Fungal Synthetic Microbial Community for Efficient Lignin Degradation

**Figure 10:**
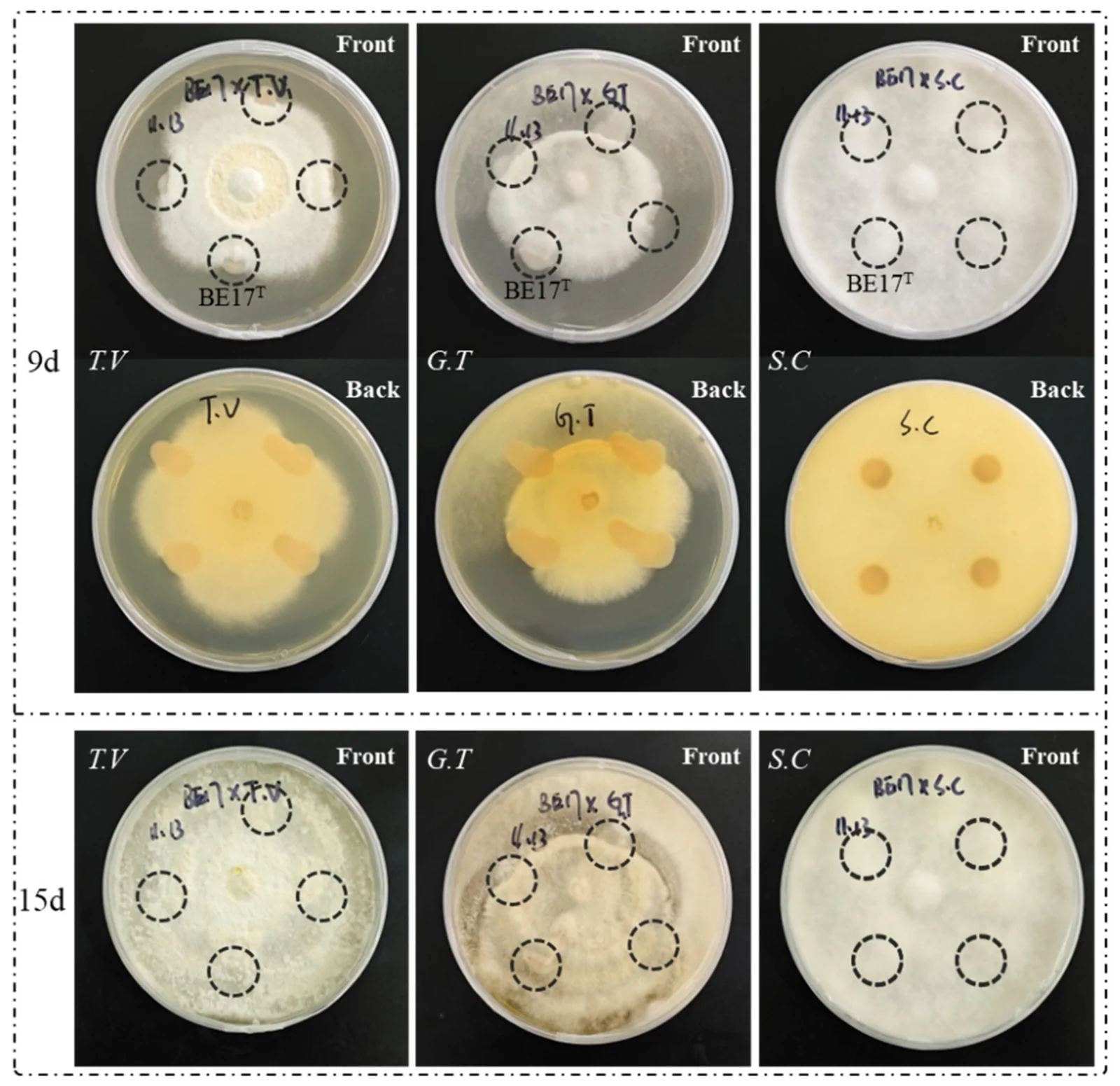
Confrontation growth of strain BE17^T^ and decaying fungi on plates.

The strain BE17^T^ was co-cultured with three types of decay fungi (*Trametes versicolor*, *T.V*; *Gloeophyllum trabeum*, *G.T*; *Schizophyllum commune*, *S.C*) on PDA-NA composite medium plates to analyze their antagonistic effects. The results showed that by the ninth day of cultivation, the decay fungi *T.V*, *G.T*, and *S.C* grew well on the composite medium plates, with *S.C* exhibiting the fastest growth, as its mycelium had fully covered the plate. Further observation of the front and back of the plate revealed that strain BE17^T^ showed significant growth, and no antagonistic effect was observed with any of the three fungi. After continued cultivation until day 15, all fungi had fully covered the plates, and no antagonistic inhibition was observed with strain BE17^T^ (Figure 10), indicating that the decay fungi *T.V*, *G.T*, and *S.C* can all serve as candidate fungi for constructing a synthetic microbial community with strain BE17^T^.

**Figure 11:**
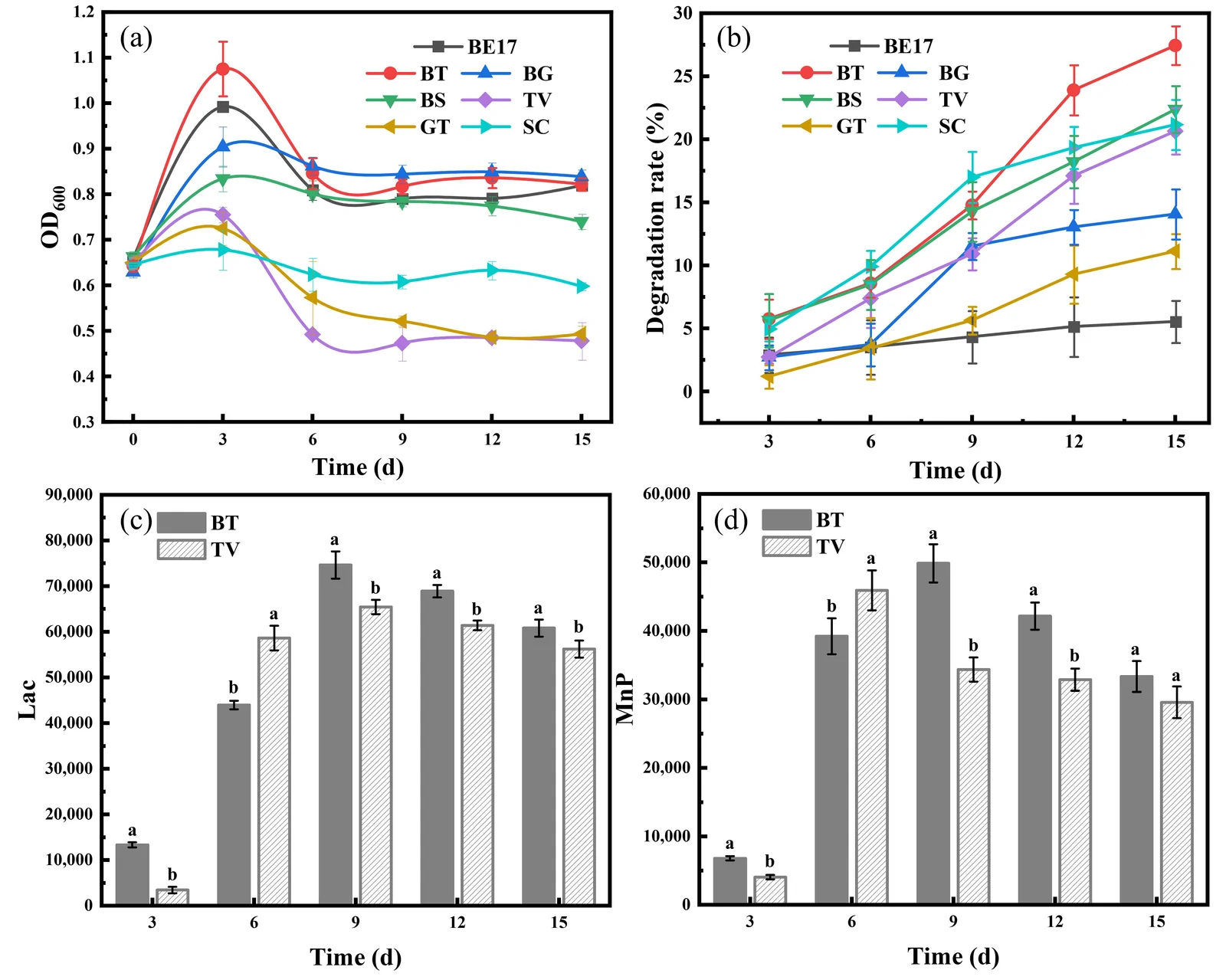
Selection of lignin-degrading composite microbial consortium. (**a**) microbial growth (OD_600_); (**b**) lignin degradation rate; (**c**) laccase activity; (**d**) manganese peroxidase activity. Different lowercase letters indicate statistically significant differences (*p* < 0.05) between groups.

The single bacterium strain BE17^T^; single fungi *T.V*, *G.T*, *S.C*; and the synthetic microbial communities without antagonistic inhibition (BE17^T^+*T.V*, BT; BE17^T^+*G.T*, BG; BE17^T^+*S.C*, BS) were separately inoculated into lignin degradation media. Samples were taken every 3 days to measure growth, lignin degradation rate, and enzyme activity. The results showed that during the first 3 days of shaking culture, the OD_600_ value of the group inoculated with BE17^T^ increased significantly, while the OD_600_ value of the single-fungus groups increased slightly (Figure 11a). This is because the decaying fungi *T.V*, *G.T*, and *S.C* are filamentous fungi, which form mycelial pellets during liquid culture [107]. The OD_600_ value (optical density) reflects the cell density suspended in the culture liquid, and a higher OD600 value indicates a higher biomass in the culture [108]. Therefore, at this stage, the OD_600_ value primarily reflects the growth of strain BE17^T^. The OD_600_ value increased rapidly in the first 3 days and gradually stabilized after day 6, indicating that the growth and metabolic cycle of strain BE17^T^ primarily occurred in the first 6 days. Additionally, compared with single-bacterium BE17^T^, the synthetic microbial community with decaying fungus *T.V* (BT) showed the highest growth on day 3, indicating that the inclusion of decaying fungus *T.V* promoted the growth of strain BE17^T^ to some extent. Some studies have shown that cell biomass yield can be increased by co-culture of bacteria and fungi, and each strain can promote the growth of another strain [109,110]. Lignin degradation rate measurements showed that the degradation rate of single-bacterium BE17^T^ after 15 days in lignin as the sole carbon source liquid medium was only 5.6%, while the degradation rate of single-fungus *T.V* was 20.7%, and the degradation rate of the BT synthetic microbial community reached 27.4%, significantly improving its degradation performance (Figure 11b). Similarly, Zhao et al. [36] found that the alkaline lignin degradation efficacy of strain combinations of *Pseudomonas oleovorans* XKG6, *Clonostachys compactiuscula* LCN1, and *Paracremonium* sp. LCB1 significantly surpassed that of individual strains. Lignin breakdown is promoted by the collaborative interaction of various fungal and bacterial species in natural environments [111]. The co-culturing approach of fungi and bacteria has been shown to effectively decolorize dye effluent [26] and degrade polycyclic aromatic hydrocarbons [112]. The enhanced microbial activity observed in co-culture is likely attributable to synergistic interactions between strain BE17^T^ and *T. versicolor* in this study. Accordingly, co-cultivating fungi and bacteria at an appropriate ratio can boost microbial growth and enhance cell viability. Consequently, the optimal combination comprising *T.V* and strain BE17^T^ were selected for subsequent investigations.

Enzyme activity analysis of the fermentation supernatant from each group revealed that laccase and manganese peroxidase activities were detected only in the group inoculated with decaying fungus *T.V*, while weak or no activity was detected in other groups. This indicates that the enzyme activity in the BT fermentation liquid mainly comes from the decaying fungus *T.V*. The maximum laccase and manganese peroxidase activities in the BT synthetic microbial community were higher than those in single-fungus *T.V* (Figure 11c,d). Consistent with the observation by Kamei [113], the present result shows that *T. versicolor* produces more laccase when grown together with bacterial strains *Cupriavidus* sp. TN6W-26 or *Enterobacter* sp. TN6W-26. Bidirectional signal transmission is essential for fungal–bacterial co-culture systems to achieve functional synergy. Chemical signals released by bacteria may regulate fungal gene expression, thereby influencing fungal enzymatic activity [114]. In *Phanerochaete chrysosporium*, indole at concentrations of 10–50 μM was shown to increase manganese peroxidase (MnP) activity by around 1.8 times, which facilitated the breakdown of polycyclic aromatic hydrocarbons [115]. Therefore, we speculate that during the early growth of strain BE17^T^, certain metabolites, potentially including low-molecular-weight phenolic compounds, indole derivatives, or autoinducer-like molecules, may have been produced. These metabolites could act as signaling molecules to induce the expression of lignin-degrading enzyme genes in *T. versicolor*, extending its active metabolic period. However, since the GC-MS analysis was conducted on the bacterial monoculture rather than the co-culture system, the specific identity of the signaling molecules and the underlying regulatory mechanism remain to be elucidated. Future studies employing comparative metabolomics and transcriptomics of the co-culture system will be required to validate this hypothesis.

### 3.8. Optimization of Cultivation Conditions for the Synthetic Microbial Community in Lignin Degradation Analysis

#### 3.8.1. The Effect of Different pH Levels on Lignin Degradation by the Synthetic Microbial Community

**Figure 12:**
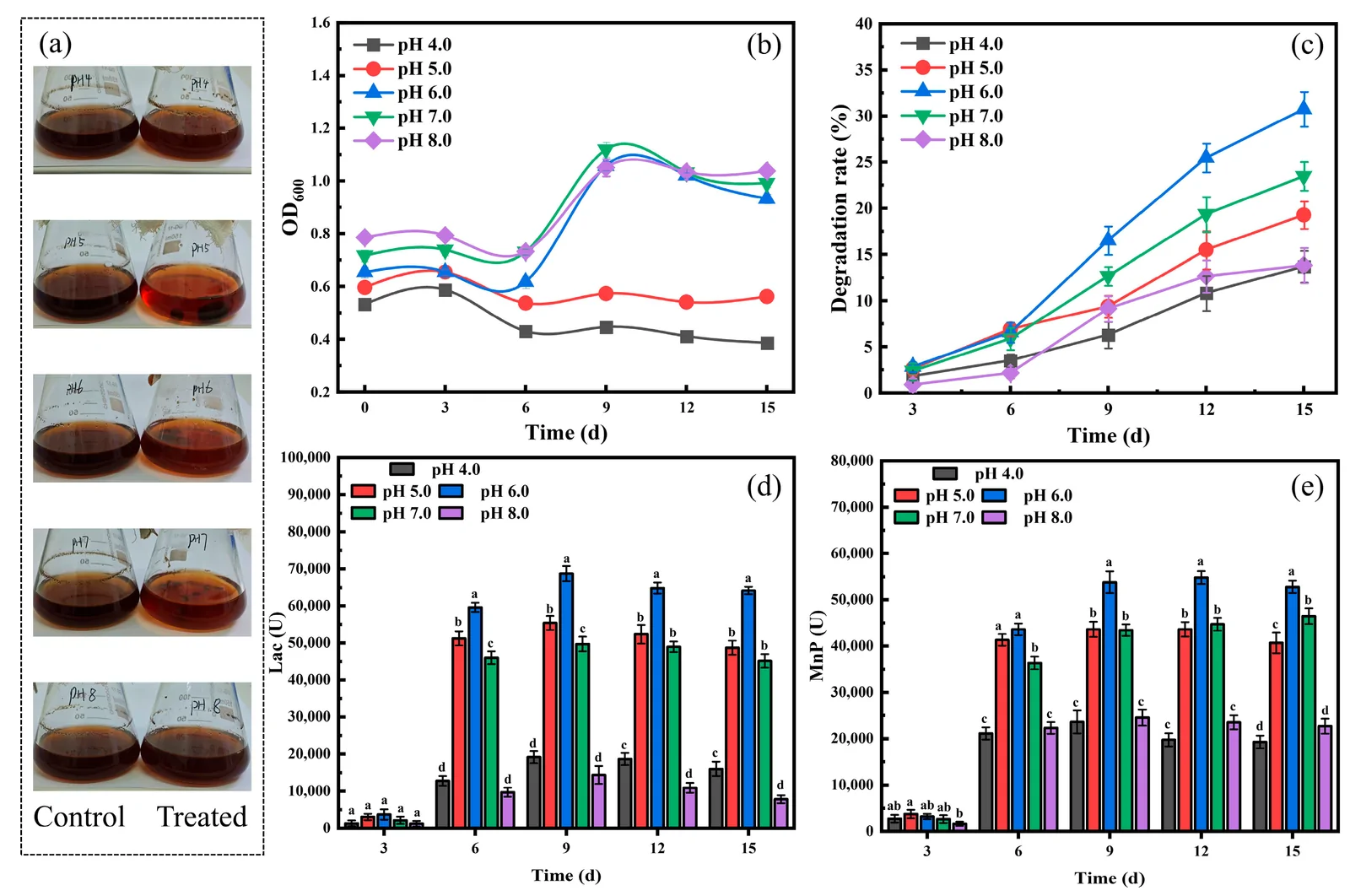
Differences in lignin degradation performance of the BT composite microbial consortium under different pH incubation conditions: (**a**) lignin degradation liquid in flasks; (**b**) microbial growth; (**c**) lignin degradation rate; (**d**) laccase activity; (**e**) manganese peroxidase activity. Different lowercase letters indicate statistically significant differences (*p* < 0.05) between groups.

Due to differences in the optimal pH conditions for bacterial and fungal growth, a series of media with different pH values (4.0, 5.0, 6.0, 7.0, and 8.0) were prepared to determine the optimal pH for the lignin degradation by the synthetic microbial community. Samples were taken regularly to measure microbial biomass, lignin degradation rate, and enzyme activity in order to analyze the effect of pH on lignin degradation by the synthetic microbial community. After 15 days of degradation by the BT synthetic microbial community, the lignin-containing medium in flasks under pH 6.0, 7.0, and 8.0 showed a visibly lighter color, whereas little change was observed at pH 4.0 and 5.0 (Figure 12a). On day 6, after the addition of strain BE17^T^, OD_600_ values increased significantly under pH 6.0, 7.0, and 8.0, while no obvious increase was observed at pH 4.0 and 5.0, indicating that strain BE17^T^ could not grow well under acidic conditions below pH 5.0 (Figure 12b). Additionally, the lignin degradation rate under pH 6.0 reached 30.7%, significantly higher than that under other pH conditions (Figure 12c). Laccase and manganese peroxidase activities showed the same trend, with the highest enzyme activities observed under pH 6.0, both peaking on day 9 (Figure 12d,e).

The degradation of lignin by *Trametes versicolor* is significantly influenced by the extracellular pH, which regulates the activity of its lignin-modifying enzyme system and the overall degradative process. Research indicates that an acidic to near-neutral pH range is generally optimal, with specific optima varying depending on the target substrate, the specific fungal strain, and the enzyme being measured. For instance, in a study optimizing conditions for a *T. versicolor* strain (BYL-7) degrading alkaline lignin and rice straw, response surface methodology identified an initial pH of 6.7 as optimal, resulting in a lignin degradation rate of 36.5% and peak activities of laccase (Lac), lignin peroxidase (LiP), and manganese peroxidase (MnP) [116]. This suggests that for comprehensive lignocellulose breakdown, a moderately acidic condition may be favorable. The effect of pH is directly mediated through its impact on the stability and catalytic efficiency of key extracellular enzymes. Laccase, a multicopper oxidase crucial for lignin modification, from *T. versicolor* typically exhibits its highest activity in acidic conditions. One study on a purified laccase (LAC1) from *T. versicolor* HBB 7328 reported an optimum activity at pH 5.0, with the enzyme remaining stable in a broad range from pH 3.0 to 7.0 [117]. The sensitivity of laccase to pH is rooted in its structure; the ionization state of residues near the catalytic copper center and the substrate itself affect electron transfer.

#### 3.8.2. Effect of Different Incubation Temperatures on Lignin Degradation by the Synthetic Microbial Community

**Figure 13:**
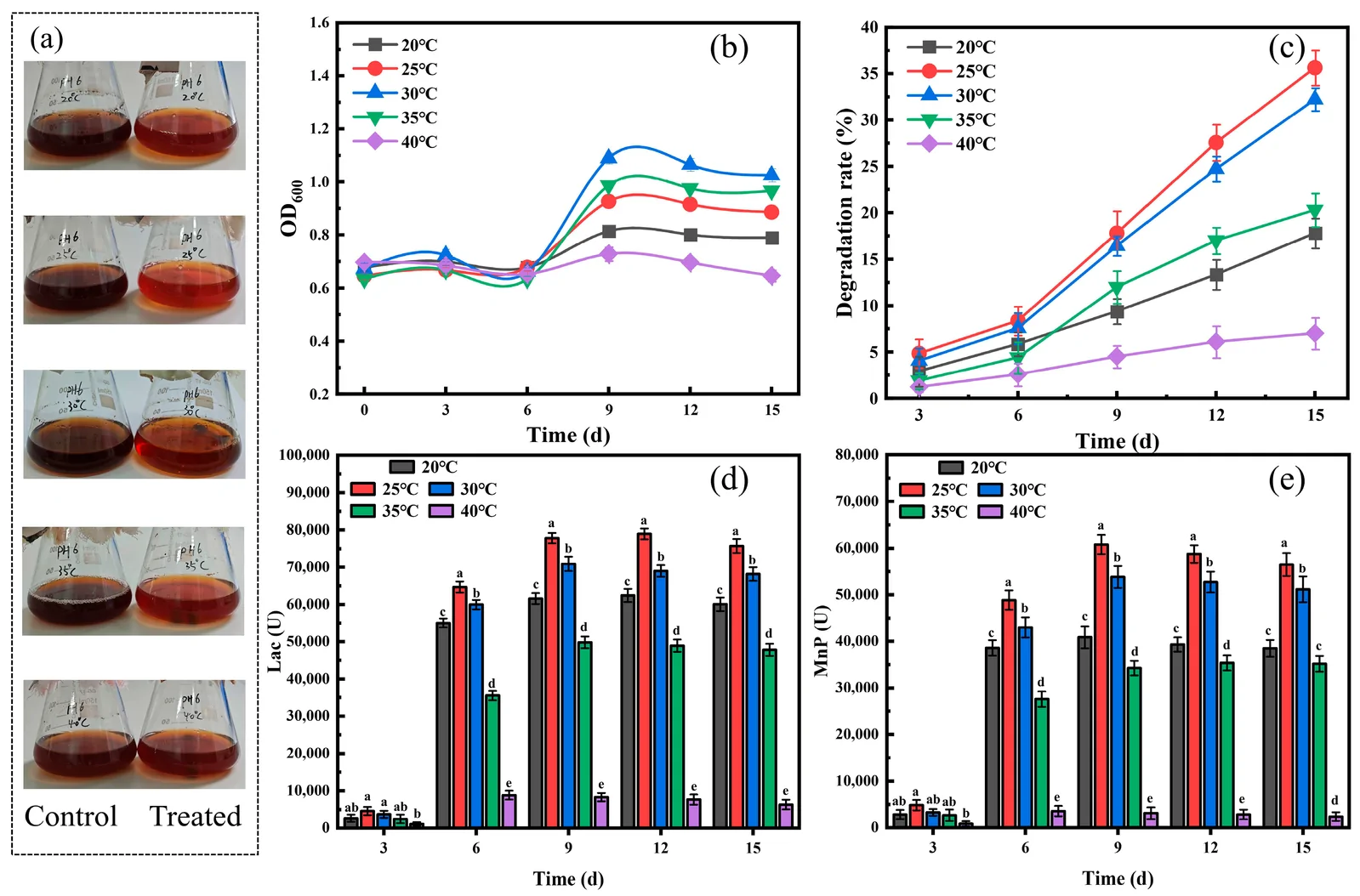
Differences in lignin degradation performance of the BT composite microbial consortium under different incubation temperatures: (**a**) lignin degradation liquid in flasks; (**b**) microbial growth; (**c**) lignin degradation rate; (**d**) laccase activity; (**e**) manganese peroxidase activity. Different lowercase letters indicate statistically significant differences (*p* < 0.05) between groups.

After 15 days of incubation under different temperature conditions, microbial growth, lignin degradation rate, and degradation enzyme activities were measured. The results showed that temperature is one of the key environmental factors affecting the lignin degradation efficiency of the synthetic microbial community, as it not only determines microbial growth activity but also regulates the expression level and catalytic efficiency of related enzymes. As shown in Figure 13a–c, the synthetic microbial community BT exhibited the best lignin degradation performance at 25 °C and 30 °C, with degradation rates of 35.6% and 32.2%, respectively. Under 20 °C conditions, the activity of strain BE17^T^ was inhibited, leading to reduced nutrient uptake and metabolic rates, which, in turn, affected cell proliferation and enzyme expression, reflected by relatively low OD_600_ values and lignin degradation rates. In contrast, temperatures between 25 and 30 °C provided a moderate and favorable environment for both bacterial and fungal metabolic activity, promoting hyphal expansion and rapid cell proliferation. However, at a high temperature of 40 °C, strain BE17^T^ likely experienced heat stress, showing signs of growth inhibition or even cell death, resulting in no significant changes in the OD_600_ value of the culture. Additionally, laccase and manganese peroxidase activities were also significantly affected by incubation temperature (Figure 13d,e).

Incubation temperature exerts a profound and multi-faceted influence on the lignin degradation efficacy of *Trametes versicolor*, primarily by regulating the fungal growth rate, the production of lignin-modifying enzymes, and the activity and stability of the enzymes themselves. Research indicates that *T. versicolor* can grow and produce key ligninolytic enzymes such as laccase and manganese peroxidase (MnP) across a broad temperature spectrum, from as low as 5 °C to 35 °C [118]. However, the optimal temperature for maximal enzyme production is distinct from the optimal temperature for the activity of the purified enzymes. Studies have shown that the highest production of laccase and MnP by *T. versicolor* occurs at 35 °C [119]. Conversely, other studies optimizing conditions for specific strains and substrates, such as rice straw, have identified a lower optimal temperature of 25 °C for overall lignin degradation [116]. This variability suggests that the ideal temperature for the degradative process can be strain-dependent and influenced by other culture conditions, including the substrate composition and pH. Temperature affects the accessibility of lignin to enzymatic attack through multiple mechanisms, including changes in substrate swelling, solubility, and molecular organization. These physical and chemical changes can significantly influence the efficiency of lignin degradation by *Trametes versicolor*.

#### 3.8.3. Effect of Different Bacterial Inoculation Times on Lignin Degradation by the Synthetic Microbial Community

**Figure 14:**
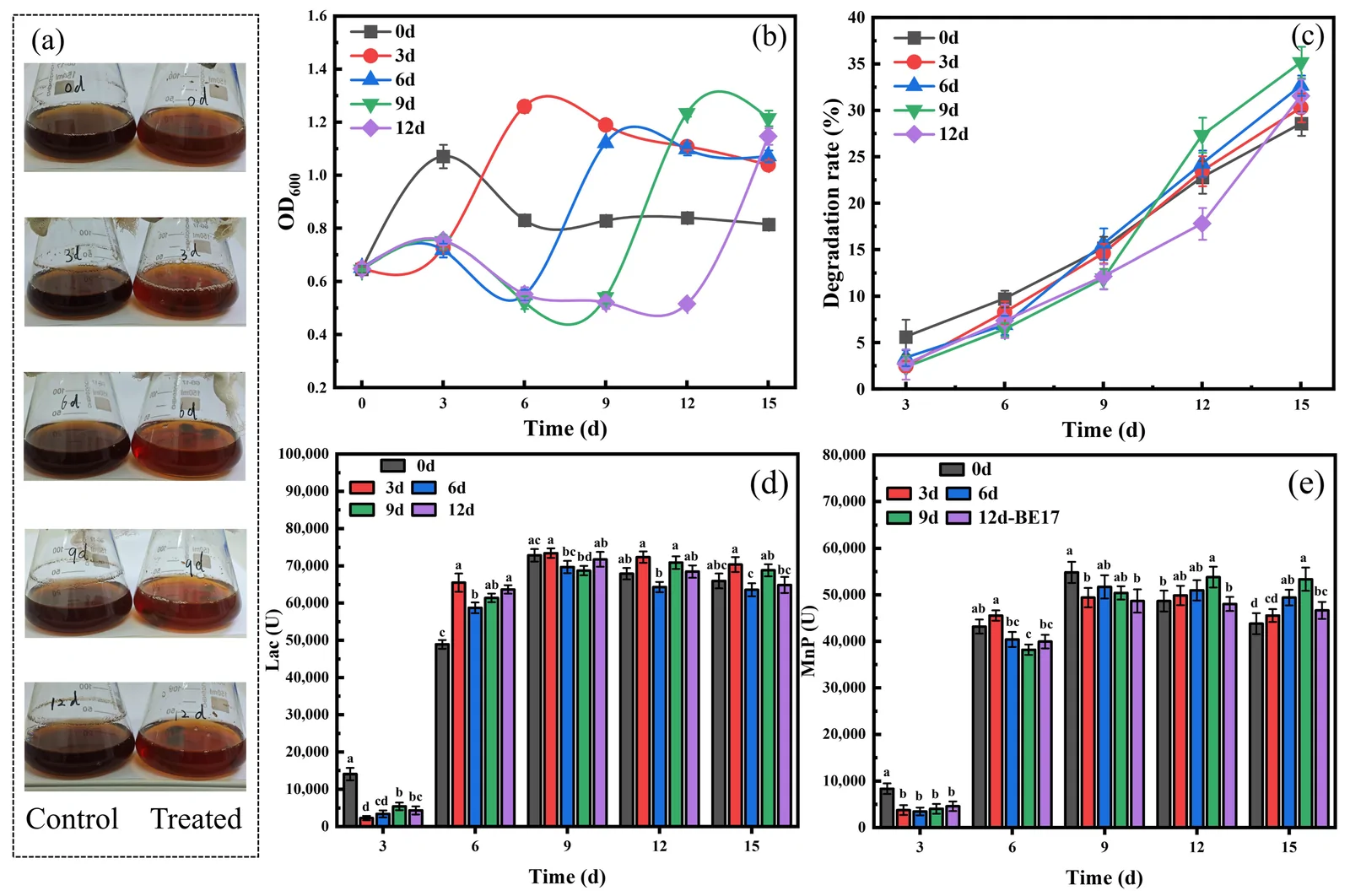
Differences in lignin degradation performance of the BT composite microbial consortium at different bacterial inoculation times: (**a**) lignin degradation liquid in flasks; (**b**) microbial growth; (**c**) lignin degradation rate; (**d**) laccase activity; (**e**) manganese peroxidase activity. Different lowercase letters indicate statistically significant differences (*p* < 0.05) between groups.

To elucidate the temporal interaction mechanism between bacteria and fungi and thereby optimize their synergistic efficiency in the degradation of complex substrates, strain BE17^T^ was inoculated into the culture system on days 0, 3, 6, 9, and 12, respectively (Figure 14a). Samples were taken at regular intervals to measure microbial biomass, lignin degradation rate, and enzymatic activity. The results showed that when strain BE17^T^ was introduced after a period of pre-cultivation with fungus *T.V*, its biomass was generally higher than that in groups where the bacterium and fungus were inoculated simultaneously (Figure 14b). This effect was most pronounced when BE17^T^ was added on day 9. This may be attributed to the fact that fungus *T.V* had already depolymerized part of the lignin into low-molecular-weight intermediates during the early stage of cultivation, providing a more favorable nutritional basis for subsequent bacterial growth and metabolism. At this stage, the fungus had preliminarily established a metabolically advantageous environment, which the bacteria could then exploit to proliferate rapidly and participate in further transformation processes. In addition, differences in lignin degradation rates also reflected this temporally optimized synergistic interaction. Compared with simultaneous inoculation, groups in which BE17^T^ was added at later stages exhibited higher lignin degradation rates (Figure 14c), with the highest rate (35.2%) observed when the strain was introduced on day 9, indicating that the bacterium demonstrated greater degradation efficiency toward the products pretreated by the fungus. Interestingly, the activity of manganese peroxidase showed a similar trend, whereas activity of laccase showed little effect of bacterial inoculation time (Figure 14d,e).

The biological rationale for this optimized timing is rooted in the distinct biochemical roles of fungi and bacteria in lignin modification. *Trametes versicolor* employs extracellular peroxidases and laccases to attack the robust lignin polymer, a process that often leads to partial mineralization and the release of solubilized, lower-molecular-weight phenolic compounds [119,120]. Bacteria are typically inefficient at breaking down intact lignin but excel at utilizing these soluble aromatic metabolites as carbon sources [95]. Therefore, inoculating bacteria too early, before the fungal enzymes have generated sufficient metabolites, may force bacteria to compete with the fungus for simpler, more accessible nutrients (e.g., sugars or nitrogen), potentially stifling fungal growth and ligninolytic enzyme production. Conversely, inoculating bacteria at the optimal window, once the fungal oxidative machinery is active, ensures they efficiently channel the lignin-derived compounds into central metabolism, preventing the potential re-polymerization of phenolics and driving the degradation process forward. In complex bioprocesses like composting, research has demonstrated that a multi-stage inoculation strategy, where specific microbial consortia are added at different phases, can optimize the community structure and functional diversity, thereby enhancing the overall degradation of organic matter including lignocellulose [121]. This principle applies directly to engineered consortia for lignin breakdown, where premature bacterial introduction can lead to competitive inhibition for resources, while delayed introduction may miss key synergistic windows for metabolizing fungal-derived lignin fragments.

#### 3.8.4. Validation of the Optimal Performance of the Lignin-Degrading Microbial Consortium

**Figure 15:**
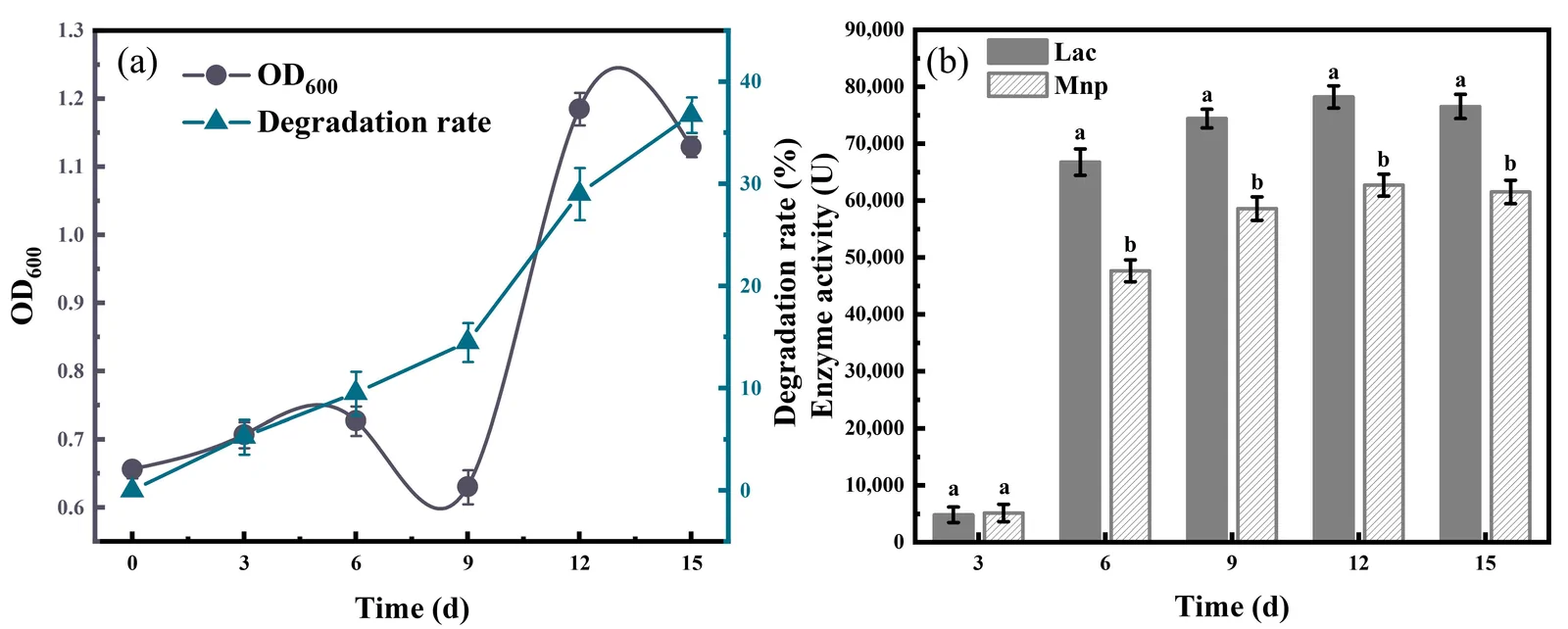
Verification of lignin degradation capability of the BT consortium under optimized culture conditions: (**a**) microbial growth and lignin degradation rate; (**b**) lignin degradation enzyme activity. Different lowercase letters indicate statistically significant differences (*p* < 0.05) between groups.

To verify the enhancing effect of the optimal culture conditions obtained from the single-factor optimization experiments on the lignin-degrading performance of the microbial consortium, the following parameters were set: pH 6.0, incubation temperature 25 °C, inoculation amount of the white-rot fungus *T.V* at 2%, shaking speed 150 rpm. After a 9-day pre-incubation of *T.V*, strain BE17^T^ was inoculated at the same inoculum level, and the culture was continued for another 6 days. The results showed that during the initial 0–9 days of fungal monoculture, the lignin degradation rate exhibited a steady upward trend, while the OD_600_ increased slightly in the first 6 days, and then declined from day 6 to day 9 (Figure 15a). This is because, in the early stage of liquid fermentation, *T.V* propagates via sporulation, generating a large number of free spores that remain well suspended, leading to a rising OD_600_. As the culture progresses, *T.V* transitions to hyphal growth, forming filamentous mycelial pellets that settle, reducing the number of suspended cells, and thus, causing a decline in OD_600_ [122]. After the inoculation of BE17^T^ on day 9, the OD_600_ of the culture system increased rapidly, indicating vigorous bacterial proliferation. Simultaneously, the lignin degradation rate significantly improved, reflecting the efficient utilization of fungal degradation intermediates by the bacteria. By day 15, the lignin degradation rate reached 36.7%, markedly higher than the 27.4% under unoptimized conditions, confirming the effectiveness of the optimization strategy. Furthermore, the maximum activities of laccase and manganese peroxidase reached 76,543 U and 62,524 U, respectively, indicating enhanced enzyme activity of the fungal system under optimized conditions (Figure 15b). The synergistic interaction with the bacteria may also positively influence the expression and secretion of fungal extracellular enzymes [109,123]. In summary, the optimized culture conditions significantly improved the lignin degradation efficiency of the microbial consortium.

However, as the co-culture system was newly established and the optimal ranges for pH, temperature, and bacterial inoculation time were unknown, we only employed single-factor experiments to explore the optimal condition. This approach allowed us to independently evaluate the effect of each parameter on lignin degradation performance before undertaking more complex multifactorial designs. We acknowledge that response surface methodology (RSM) or central composite design would provide more comprehensive optimization by identifying factor interactions, and this will be pursued in future studies.

### 3.9. Analysis of the Synergistic Enhancement Mechanism in Lignin Degradation by the Synthetic Microbial Community

**Figure 16:**
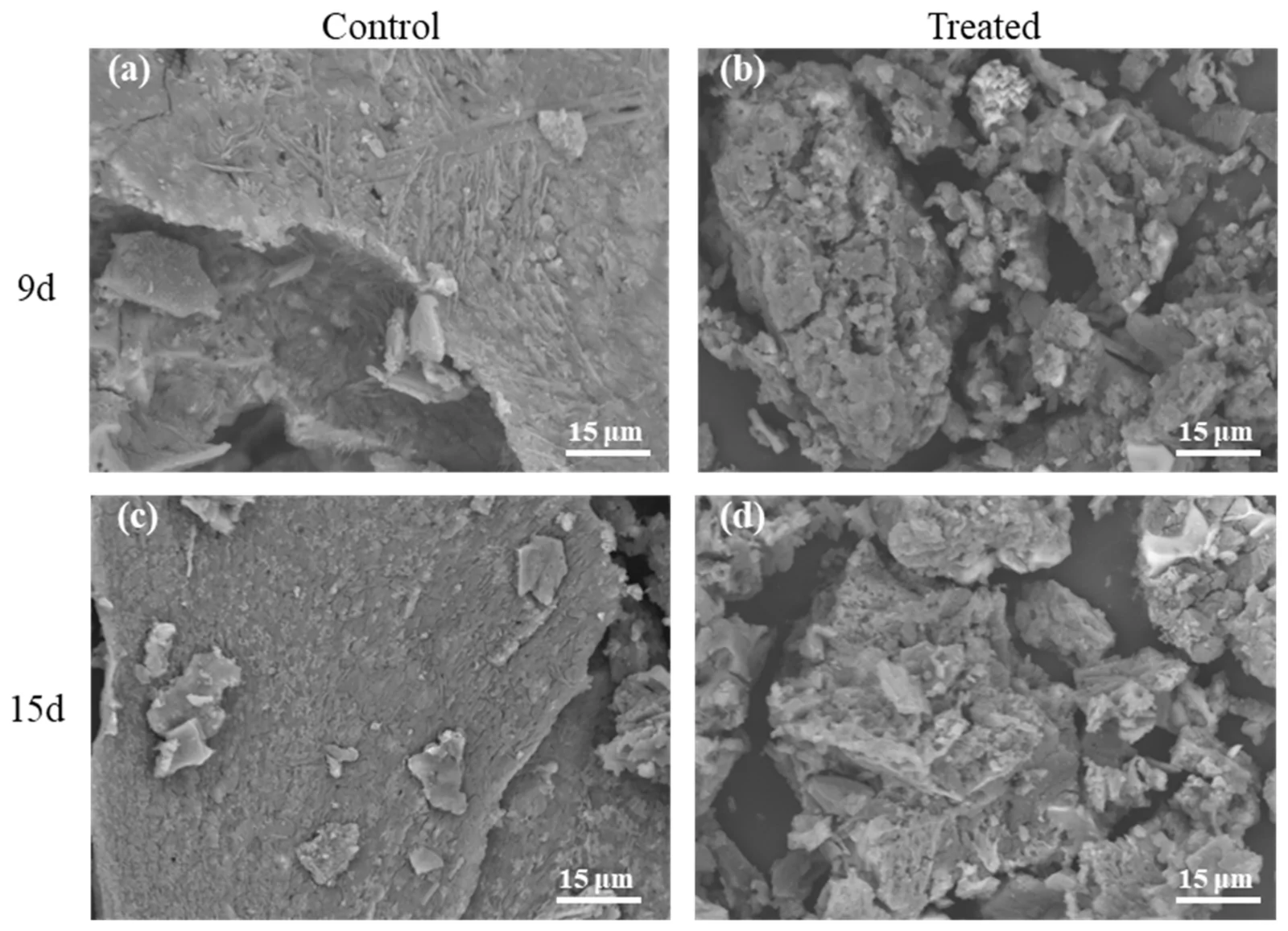
Micromorphological changes during lignin degradation by the BT consortium. Scanning electron micrograph of untreated (**a**) and BT consortium treated (**b**) lignin after 9 days; Scanning electron micrograph of untreated (**c**) and BT consortium treated (**d**) lignin after 15 days.

To elucidate the potential synergistic mechanism of the BT microbial consortium during lignin degradation, scanning electron microscopy (SEM) and Fourier-transform infrared spectroscopy (FTIR) analyses were employed on lignin powder collected before the addition of strain BE17^T^ (day 9) and after 15 days of cultivation, including both control and treatment groups. The results showed that there were no significant morphological differences in lignin between the day 9 and day 15 control groups, indicating that in the absence of microbial activity, the external structure of lignin remained relatively stable. In contrast, the lignin surfaces from the day 9 and day 15 treatment groups both exhibited clear signs of structural damage, such as increased surface roughness, porosity, and cracking (Figure 16), demonstrating that microbial treatment played a crucial role in lignin depolymerization. Notably, there were minimal morphological differences between the lignin samples from the day 9 and day 15 treatment groups, suggesting that the majority of structural degradation occurred during the first 9 days. This indicates that the extracellular laccase and manganese peroxidase secreted by the white-rot fungus *T.V* during the early phase were primarily responsible for significant structural disruption of lignin. The subsequently added strain BE17^T^ primarily contributed through intracellular enzymatic activity, metabolizing and transforming the resulting low-molecular-weight degradation products.

**Figure 17:**
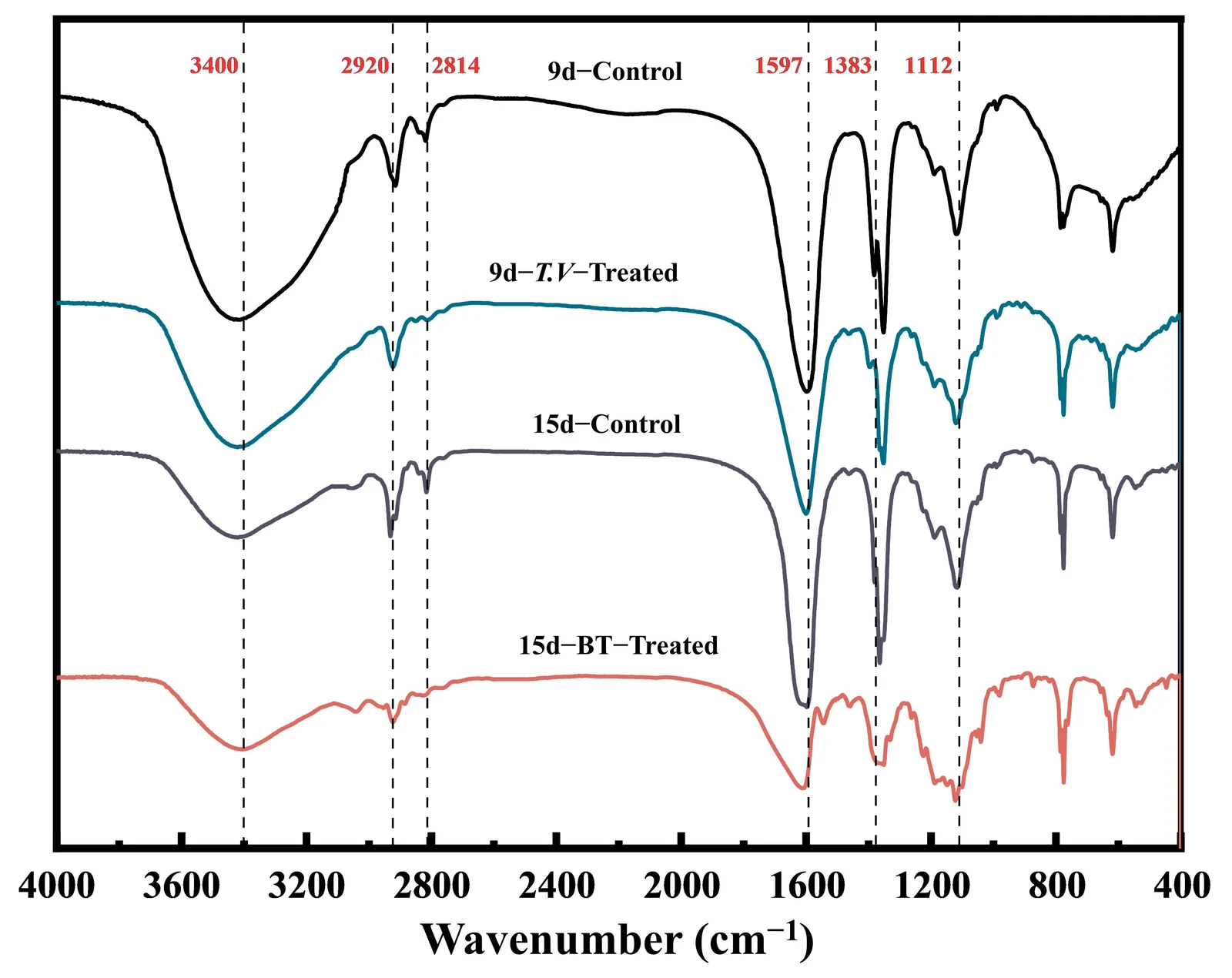
Chemical structural changes in lignin degradation by the BT revealed by FTIR.

Further FT-IR spectroscopy analysis revealed significant changes in the chemical structure of lignin during the degradation process (Figure 17). After a 9-day treatment with the white-rot fungus *T.V*, the absorption intensities at 3400 cm^−1^ (–OH stretching vibration), 2920 cm^−1^ (C–H asymmetric stretching), 1597 cm^−1^ (aromatic skeletal vibration), 1384 cm^−1^ (aromatic C–H deformation), and 1112 cm^−1^ (C–O–C stretching) were all weakened compared with the untreated control group. Notably, the absorption peak at 2814 cm^−1^ (C–H symmetric stretching) almost disappeared, indicating that *T.V* depolymerized lignin by secreting extracellular enzymes such as laccase and manganese peroxidase, which attacked the ether bonds in lignin and further disrupted phenolic hydroxyl groups, methoxy groups, aliphatic side chains, and the aromatic backbone. After inoculation with strain BE17^T^ on day 9 and an additional 6 days of incubation, the lignin structure was further degraded. Compared with the 9-day treatment group, the absorption peaks at 2920 cm^−1^ and 1597 cm^−1^ in the 15-day treatment group significantly decreased, while the peaks at 2814 cm^−1^ and 1384 cm^−1^ completely disappeared. Meanwhile, the 1112 cm^−1^ peak also declined. These results suggest that, under bacterial activity, lignin-derived monomers and side chains were further oxidized and degraded, and they likely entered the bacterial intracellular metabolic pathway for further transformation.

**Figure 18:**
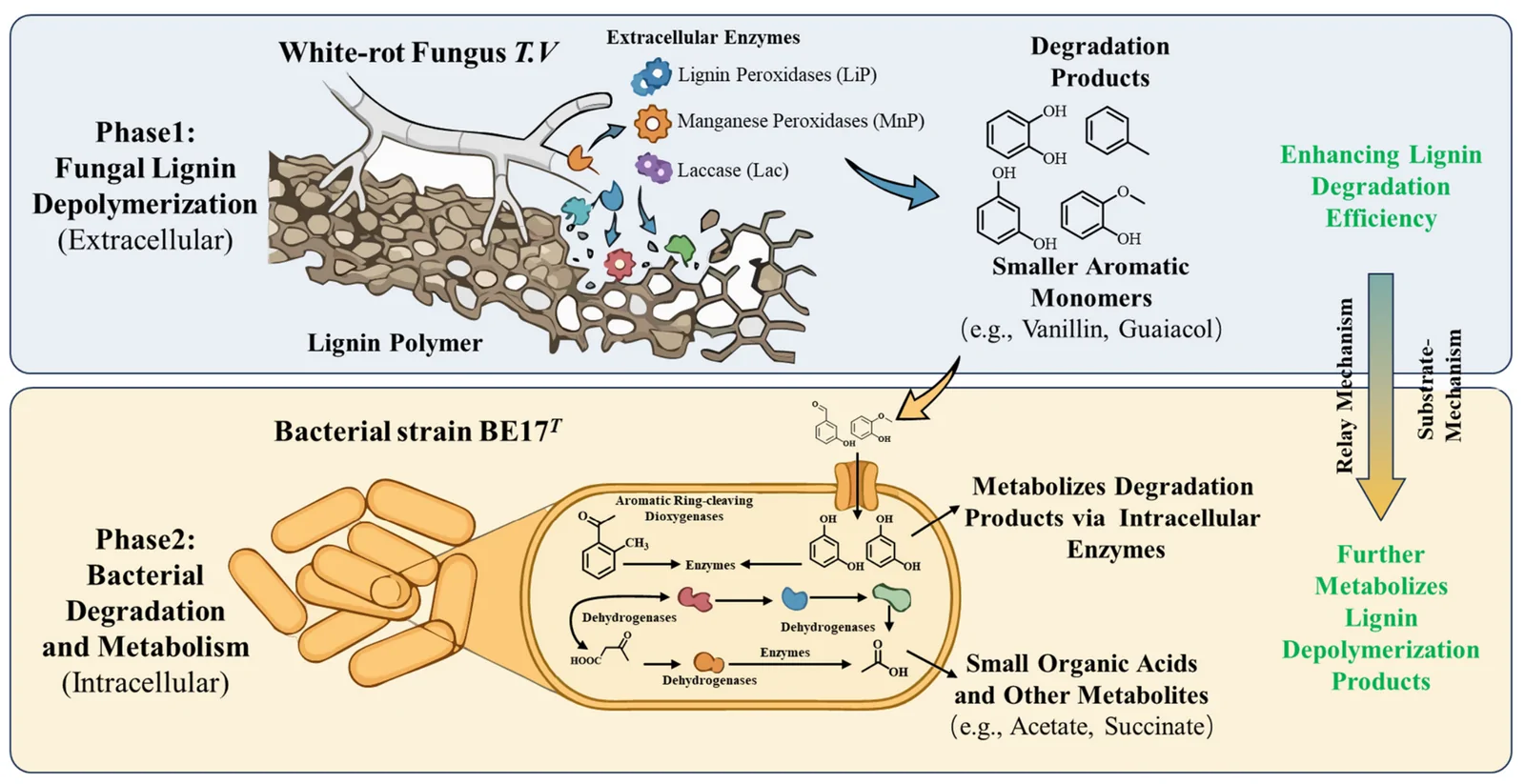
A hypothetical model for the substrate–metabolism relay mechanism of the microbial consortium BT, based on the current findings and previous reports.

In summary, the synergistic mechanism between strain BE17^T^ and white-rot fungus *T.V* is likely a substrate–metabolism relaying mechanism (Figure 18). *T.V* first depolymerizes lignin into smaller aromatic monomers via extracellular enzymes, and strain BE17^T^ subsequently metabolizes these degradation products via intracellular enzymes, converting them into small organic acids or other metabolites. This process not only enhances lignin degradation efficiency but also highlights the critical role of strain BE17^T^ in further metabolizing the lignin depolymerization products produced by the fungus.

## 4. Conclusions

In this study, a novel lignin-degrading bacterium, *Brucella ligniniphila* sp. nov. BE17^T^, was isolated from decaying bamboo. Lignin degradation was enhanced by glucose supplementation (from 7.9% to 17.2%). Structural analysis showed that BE17^T^ attacks β–O–4 bonds, oxidizes side-chain aliphatic structures, and disrupts aromatic rings, with low-molecular-weight lignin being more readily degraded. The strain exhibited high aniline blue decolorization (80.9%), and genome annotation revealed genes encoding dye-decolorizing peroxidase and other ligninolytic enzymes. GC-MS detected various aromatic monomers and organic acids, suggesting that BE17^T^ metabolizes lignin-derived aromatics via CoA-dependent non-β-oxidative, β-ketoadipate, and phenylacetic acid pathways. Based on these characteristics, a synthetic microbial consortium (BT) consisting of BE17^T^ and the white-rot fungus *Trametes versicolor* was constructed. The consortium achieved a degradation rate of 27.4%, significantly higher than either pure culture (5.6% and 20.7%). Under optimized conditions (25 °C, pH 6.0, bacterial inoculation on day 9), the degradation rate reached 36.7%. Microstructural and chemical analyses confirmed that fungal pretreatment facilitated subsequent bacterial degradation. This study provides insights into the ligninolytic mechanisms of a novel *Brucella* species and demonstrates a highly efficient bacterial–fungal synergistic system, offering valuable references for lignin bioconversion.

### Description of Brucella ligniniphila sp. nov

*Brucella ligniniphila* (lig.ni.ni’phi.la. N.L. neut. n. *ligninum*, lignin; N.L. masc. adj. suff. -philus, friend, loving; from Gr. masc. adj. *philos*, loving; N.L. fem. adj. *ligniniphila*, lignin loving).

Strain BE17^T^ is a Gram-negative, non-motile, short rod-shaped bacterium (0.6–0.7 × 0.9–1.1 µm) with fimbriae but lacking flagella. After 48 h of incubation on LB agar, colonies were 0.5–0.8 mm in diameter, round, and milky white. The strain grows within the temperature range of 4–40 °C, salinity range of 0–7%, and pH range of 5.0–9.0, with optimal growth at 28 °C, 1% NaCl, and pH 8.0, demonstrating tolerance to alkaline and saline conditions. Methyl red and Voges–Proskauer (V-P) tests were negative. The strain tested positive for oxidase, catalase, hydrogen sulfide (H_2_S) production, nitrate reduction, and nitrite reduction. Weak positive reactions were observed for Tween 60 and aesculin hydrolysis, whereas negative results were obtained for the hydrolysis of Tween (20, 40, and 80), starch, carboxymethyl cellulose (CMC), tyrosine, and casein. Strain BE17^T^ demonstrated acid production from various carbon sources, including mannitol, L-arabinose, D-xylose, D-salicin, methyl-β-D-xylopyranoside, D-galactose, D-glucose, D-fructose, D-mannose, mannitol, sorbitol, cellobiose, xylitol, D-lyxose, D-tagatose, D-fucose, L-fucose, D-arabitol, and L-arabitol. Additionally, it utilized D-fructose, D-galactose, D-fucose, L-fucose, D-arabitol, D-serine, L-aspartic acid, D-galacturonic acid, L-galactonic acid lactone, D-glucuronic acid, glucuronamide, methyl pyruvate, α-ketoglutaric acid, D-malic acid, L-malic acid, and acetic acid as sole carbon sources. The predominant respiratory quinone was Q-10. The major cellular fatty acids were Summed Feature 8 (C_18:1_ *ω*7*c*; 60.20%), followed by C_19:0_ cyclo *ω*8*c* (12.94%), C_18:1_ 2OH (12.39%), and C_16:0_ (3.63%). The polar lipid profile of strain BE17^T^ comprised phosphatidylethanolamine (PE), phosphatidylmonomethylethanolamine (PEM), phosphatidylglycerol (PG), and phosphatidylcholine (PC), along with three unidentified phospholipids (PL_1-3_), two unidentified aminolipids (AL_1-2_), two unidentified glycolipids (GL_1-2_), and four other unidentified polar lipids (L_1-2_, L_5-6_).

The type strain, BE17^T^ (MCCC 1K08845^T^ = JCM 36600^T^), was isolated from decayed bamboo collected from a bamboo forest at Zhejiang A&F University, China. The DNA G+C content of the type strain was determined to be 54.86 mol% based on whole-genome sequencing. The GenBank accession numbers for the 16S rRNA gene (1406 bp) and whole-genome sequence are PP733160, CP154467, and CP154468, respectively.

## Figures and Tables

**Table 1:** Physiological and biochemical differences between strain BE17^T^ and *B. gallinifaecis* DSM 15295^T^.

Characteristic	BE17^T^	*B. gallinifaecis* DSM 15295^T^
**Sample source**	Decaying bamboo	Feces of chicken
**Salt tolerance (%,** ***w*** **/** ***v*** **) (optimum)**	0–7 (1)	ND
**pH range (optimum)**	5.0–9.0 (8.0)	ND
**Temperature range(optimum)**	4–40 (28)	ND
**Methyl red test**	−	W
**Nitrite reduction**	+	−
**Acid production from:**		
_L_-arabinose	+	−
_D_-cellobiositol I	+	−
Methyl-β-D-xylopyranoside	+	−
_D_-mannose	+	−
_D_-mannitol	+	−
_D_-sorbitol	+	−
_D_-cellobiose	+	−
Xylitol	+	−
_D_-lyxose	+	−
_D_-tagatose	+	−
_L_-fucose	+	−
_D_-arabitol	+	−
_L_-arabitol	+	−
Erythritol	−	+
_D_-arabinose	−	+
_D_-ribose	−	+
_L_-xylose	−	+
**Carbon source utilization:**		
_D_-fructose	+	−
_D_-arabitol	+	−
Glucuronamide	+	−
Methyl pyruvate	+	−
α-D-glucose	−	+
Glycerol	−	+
_L_-alanine	−	+
_L_-glutamic acid	−	+
_L_-histamine	−	+
_L_-serine	−	+
_D_-gluconic acid	−	+
_L_-lactic acid	−	+
Bromo-succinic acid	−	+
Acetoacetic acid	−	+
**Growth tolerance:**		
Oligomycin A	+	−
Guanidine hydrochloride	+	−
Rifamycin SV	−	+
Lithium chloride	−	+
**API 20NE tests:**		
Fermentation of glucose	+	−
Assimilation of arabinose	+	−
Enzymatic hydrolysis of urea	−	+
**API ZYM tests (0–5):**		
Esterase (C4)	1	2
Tryptase	2	3
Naphthol-AS-BI-phosphate hydrolase	2	1
**Polar lipids**	PE, PG, PME, PC, PL_1–3_, GL_1–2_, AL_1–2_, L_1–2,5–6_	PE, PG, PME, PC, PL_1–2_, GL_1–2_, AL_1–2_, L_1–6_
**DNA G+C content**	54.86%	51.0%

+: Positive reaction; −: Negative reaction; W: Weak positive. The 0–5 values in the ZYM test indicate the depth of the color, which corresponds to the level of enzyme activity. ND: not determined/not detected. Abbreviations for polar lipids: PE, phosphatidylethanolamine; PG, phosphatidylglycerol; PME, phosphatidylmethylethanolamine; PC, phosphatidylcholine; PL, unidentified phospholipid; GL, unidentified glycolipid; AL, unidentified aminolipid; L, unidentified polar lipid.

**Table 2:** The differences in fatty acid profiles between strain BE17^T^ and *B. gallinifaecis* DSM 15295^T^.

Peak Name	BE17^T^	*B. gallinifaecis* DSM 15295^T^
**Saturated**		
C_16:0_	3.63	7.73
C_18:0_	1.17	3.26
**Unsaturated**		
C_19:0_ cyclo *ω*8*c*	**12.94**	**25.63**
**Hydroxy**		
C_18:1_ 2OH	**12.39**	4.61
C_18:0_ 3OH	1.23	0.15
Summed feature 3 *	2.67	3.43
Summed feature 8 *	**60.20**	**51.14**

* Summed feature 3 contains C_16:1_ *ω*7*c* and/or C_16:1_ *ω*6*c*; summed feature 8 contains C_18:1_ *ω*7*c*.

**Table 3:** Statistics of gene prediction results for strain BE17^T^.

Type	Number	Total_len	Average_len	Percentage of Genome (%)
**Gene**	3811	3,436,495	902	88.38
**CDS**	3697	3,407,808	922	87.64
**tRNA**	57	4458	78	0.11
**23S rRNA**	4	11,592	2898	0.30
**16S rRNA**	4	5924	1481	0.15
**5S rRNA**	4	440	110	0.01
**tmRNA**	1	347	347	0.01
**Misc_RNA**	44	5926	135	0.15

**Table 4:** Ligninolytic enzyme-encoding genes identified in strain BE17^T^.

No.	Gene ID	bp	Gene Name	Encode Protein
**1**	gene 3029	2217	*paaG*	2-(1,2-epoxy-1,2-dihydrophenyl)acetyl-CoA isomerase
**2**	gene 3614	1515	*aldH*	2,5-dioxopentanoate dehydrogenase
**3**	gene 1542	1584	*UbiB*	2-polyprenylphenol 6-hydroxylase
**4**	gene 3190	927	*paaC*	3-hydroxyacyl-CoA dehydrogenase
**5**	gene 1822	924	*paaH*	3-hydroxybutyryl-CoA dehydrogenase
**6**	gene 0898	1050	*paaF*	3-hydroxyisobutyryl-CoA hydrolase
**7**	gene 2886	1233	*hcaD*	3-phenylpropionate/trans-cinnamate dioxygenase
**8**	gene 2802	681	*ligK*	4-hydroxy-4-methyl-2-oxoglutarate aldolase
**9**	gene 3031	1794	*dmdC*	acyl-CoA dehydrogenase
**10**	gene 3498	1518	*tgnC*	aldehyde dehydrogenase
**11**	gene 2986	1011	*BCKDHB*	alpha-ketoacid dehydrogenase
**12**	gene 2754	1236	*stc2*, *hpbB*	aromatic ring-hydroxylating dioxygenase
**13**	gene 3450	837	*ligB*	aromatic ring-opening dioxygenase
**14**	gene 0097	1098	*nspC*	carboxynorspermidine decarboxylase
**15**	gene 2549	1503	*katE*	catalase
**16**	gene 0857	861	*catE*	catechol 2,3-dioxygenase
**17**	gene 0714	1149	*gcdG*	CoA transferase
**18**	gene 2896	1326	*Dyp_perox*	DyP-type peroxidase
**19**	gene 2060	774	*echA*	enoyl-CoA hydratase
**20**	gene 3045	1287	*puuB*	gamma-glutamylputrescine oxidase
**21**	gene 0224	486	*prx3*	glutaredoxin-dependent peroxiredoxin
**22**	gene 0455	600	*GST*	glutathione S-transferase
**23**	gene 0139	825	*trmX*	methyltransferase
**24**	gene 3579	1131	*Cu-oxidase*	multicopper oxidase
**25**	gene 1581	1143	*Cu-oxidase_2*	multicopper oxidase
**26**	gene 0094	849	*paaK*	phenylacetate-CoA oxygenase
**27**	gene 3708	1452	*paaN*	phenylacetic acid degradation bifunctional protein
**28**	gene 1102	528	*paaY*	phenylacetic acid degradation protein
**29**	gene 2074	903	*pheA2*	prephenate dehydratase
**30**	gene 1943	864	*aroE*	shikimate dehydrogenase
**31**	gene 0327	597	*SOD2*	superoxide dismutase

**Table 5:** Characterization of lignin-degrading products produced by strain BE17^T^.

No.	R. Time	Compounds	Molecular Formula	Control
**1**	7.08	2-Methoxyethanol	C_3_H_8_O_2_	−
**2**	14.81	*p*-Ethylbenzaldehyde	C_9_H_10_O	−
**3**	16.29	2-Methylbenzoic acid	C_8_H_8_O_2_	−
**4**	16.5	Hexanoic acid	C_6_H_12_O_2_	−
**5**	16.96	Acetoacetic acid	C_4_H_6_O_3_	−
**6**	17.3	3-Ketovaleric acid	C_5_H_8_O_3_	−
**7**	17.3	2-Methylacetoacetic acid	C_5_H_8_O_3_	−
**8**	17.34	Ethyl malonate	C_7_H_12_O_4_	−
**9**	18.73	Phenol	C_6_H_6_O	−
**10**	19.11	Vanillin	C_8_H_8_O_3_	+
**11**	19.53	Hexadecanoic acid	C_16_H_32_O_2_	−
**12**	19.63	Cetene	C_16_H_32_	−
**13**	19.94	Hexanedioic acid	C_6_H_10_O_4_	−
**14**	20.96	1,2-Benzenedicarboxylic acid	C_8_H_6_O_4_	+
**15**	21.7	Vanillic acid	C_8_H_8_O_4_	+
**16**	22.95	3-Methoxy-4-hydroxymandelic acid	C_10_H_12_O_5_	−
**17**	23.49	*p*-Coumaric acid	C_9_H_8_O_3_	+
**18**	23.7	Dibutyl phthalate	C_16_H_22_O_4_	+
**19**	23.89	Hexadecanoic acid, ethyl ester	C_18_H_36_O_2_	−
**20**	23.92	α-Hydroxycinnamic acid	C_9_H_8_O_3_	−
**21**	24.25	2-Hydroxy-5-methylbenzoic acid	C_8_H_8_O_3_	−
**22**	24.79	2-Tolylacetic acid	C_9_H_10_O_2_	−
**23**	26.54	2′,6′-Dihydroxyacetophenone	C_8_H_8_O_3_	−
**24**	28.79	Bis(2-ethylhexyl) phthalate	C_24_H_38_O_4_	−
**25**	30.75	1,3-Benzenedicarboxylic acid	C_8_H_6_O_4_	+
**26**	30.84	3,5-Dimethylphenol	C_8_H_10_O	−
**27**	32.92	4-Hydroxybenzyl alcohol	C_7_H_8_O_2_	−

Note: “+” indicates that the compound was also detected in the control group; “−” indicates that the compound wasn’t detected in the control group.

## Data Availability

The original contributions presented in this study are included in the article and Supplementary Materials. Further inquiries can be directed to the corresponding authors.

## Supplementary Materials

The following supporting information can be downloaded at: https://www.mdpi.com/article/10.3390/microorganisms14091923/s1, Figure S1: Phylogenomic tree of strain BE17^T^ based on TYGS analysis; Figure S2: Two-dimensional thin-layer chromatogram of the total polar lipids obtained from strains BE17^T^ (a) and *B. gallinifaecis* DSM15295^T^ (b).
