# Supplementary material for: Brucella ligniniphila sp. nov., a Novel Lignin-Degrading Bacterium from Decaying Bamboo, and Synergistic Lignin Biodegradation by Consortium with White-Rot Fungi
Source: Microorganisms. 2026 Aug 31;14(9):1923. doi: 10.3390/microorganisms14091923 (PMC13609823; doi:10.3390/microorganisms14091923)
Supplement: Supplementary file 1 [file microorganisms-14-01923-s001.zip › microorganisms-4485558-supplementary.pdf]

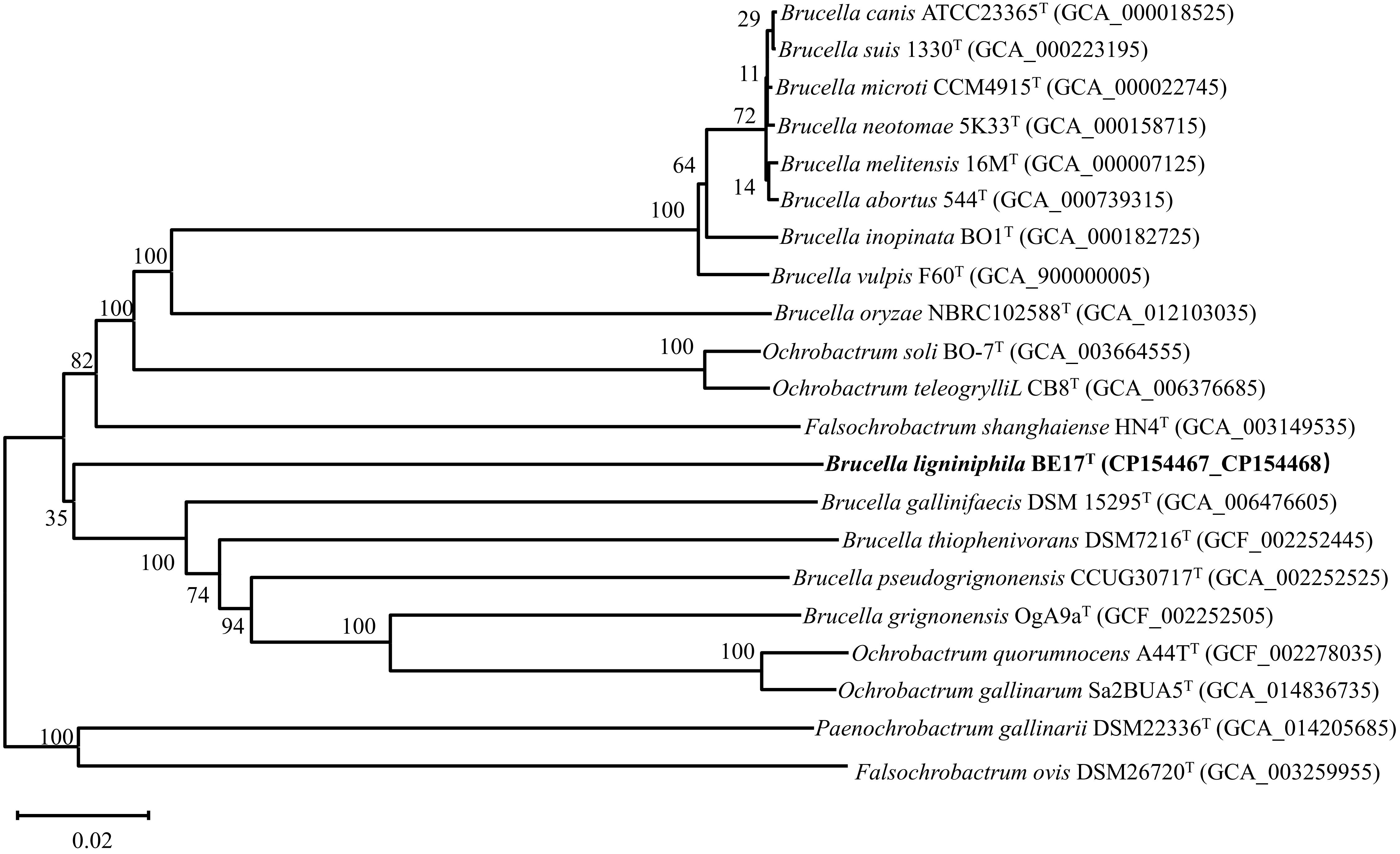

Supplementary Fig. S1. Phylogenomic tree of strain BE17^T based on TYGS analysis

Tree inferred with FastME 2.1.6.1 from GBDP distances calculated from genome sequences. The branch lengths are scaled in terms of the GBDP distance formula d_5. The numbers above branches are GBDP pseudo-bootstrap support values > 60 % from 100 replications, with an average branch support of 98.2 %. The tree was midpoint-rooted.

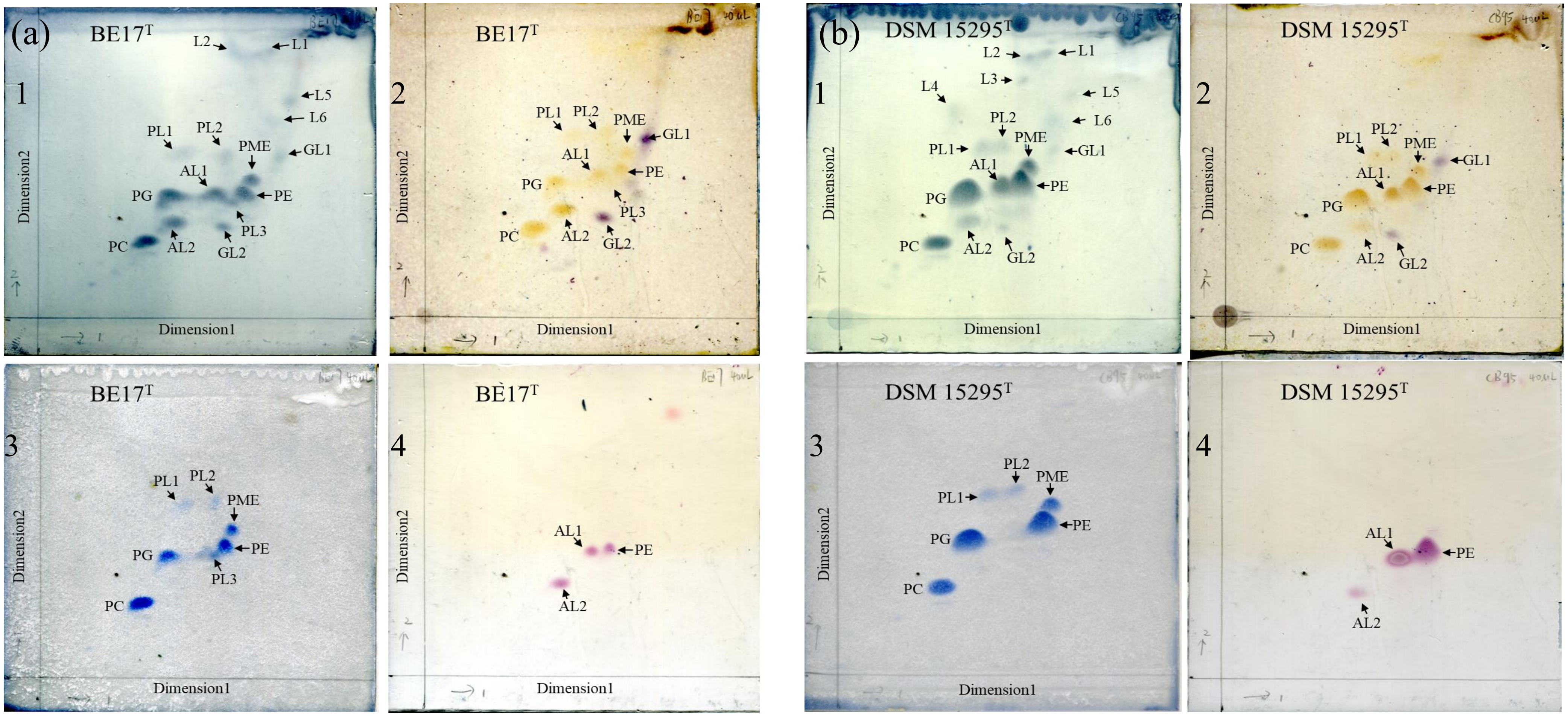

Supplementary Fig. S2. Two-dimensional thin-layer chromatogram of the total polar lipids obtained from strains BE17^{T} (a) and *B. gallinifaecis* DSM 15295^{T} (b).

The components were visualized by:

(1) 5% Molybdatophosphoric acid; (2) Alpha naphthol sulfuric acid solution; (3) Molybdenum blue (Sigma); (4) Ninhydrin.

PE, phosphatidylethanolamine; PEM, phosphatidylmonomethylethanolamine; PG, phosphatidylglycerol; PC, phosphatidylcholine; GL, glycolipid; PL, phospholipid; AL, aminolipid; L, lipid (unidentified).
